# Patient and healthcare factors associated with treatment response in patients with hand osteoarthritis: secondary analyses from a randomised controlled, noninferiority trial

**DOI:** 10.1016/j.ero.2026.100204

**Published:** 2026-06-26

**Authors:** Unni Olsen, Anne Therese Tveter, Kjetil Bergsmark, Barbara S. Christensen, Ida K. Haugen, Toril Hennig, Merete Hermann-Eriksen, Åshild Hove, Trine A. Sjøvold, Ingvild Kjeken

**Affiliations:** 1 Center for Treatment of Rheumatic and Musculoskeletal Diseases (REMEDY), Diakonhjemmet Hospital, Oslo, Norway; 2 Department of Rehabilitation Science and Health Technology, Oslo Metropolitan University, Oslo, Norway; 3 Department of Occupational Therapy, Physiotherapy, and Social Work Services, Martina Hansen Hospital, Sandvika, Norway; 4 Division of Clinical Service, Diakonhjemmet Hospital, Oslo, Norway

## Abstract

**Objectives:**

This study aimed to explore patient- and healthcare-related factors associated with treatment response 6 months after receiving recommended care for hand osteoarthritis (HOA) in specialist health services.

**Methods:**

We analysed data from 340 individuals with HOA who participated in a randomised controlled trial. Participants received patient education, medication, and/or guided hand exercises. Those with thumb carpometacarpal (CMC1-OA) were also offered a thumb orthosis. Multivariable logistic regression analyses examined associations between treatment response (OMERACT-OARSI responder criteria) and patient-related variables (age, sex, severity of CMC1-OA, pain, stiffness, grip strength, hand function, and general health), and patients’ experience with care (PasOpp questionnaire).

**Results:**

Eighty percent of participants were women, and the mean age was 63.6 years. After 6 months, 96 participants (28%) were classified as OMERACT-OARSI responders. Less severe CMC1-OA (Kellgren-Lawrence grade <2) was associated with nearly doubled odds of being a responder (odds ratio [OR] 1.95, 95% CI: 1.07-3.55). Higher baseline pain intensity also increased the likelihood of good response (OR 1.18, 95% CI: 1.05-1.33). Regarding care-related factors, high scores on ‘confidence in health professionals' competence' (OR 2.09, 95% CI: 1.10-3.96) and ‘perceived benefit of treatment’ (OR 1.74, 95% CI: 1.28-2.36) were positively associated with good response. Unexpectedly, higher scores on ‘sufficient information about diagnosis and treatment’ were associated with a lower probability of good response (OR 0.63, 95% CI: 0.42-0.96).

**Conclusions:**

Findings highlight the importance of early, individualized care and suggest that providing too much detailed information may unintentionally reduce patients’ hope for improvement.

**WHAT IS ALREADY KNOWN ON THIS TOPIC**

There is limited knowledge of how patient-related and healthcare-related factors influence treatment outcomes in hand osteoarthritis (HOA)

**WHAT THIS STUDY ADDS**

• Patients with less severe HOA and higher levels of pain were more likely to experience a positive treatment response.
• Greater trust in healthcare providers' competence and higher perceived treatment benefit was associated with better outcomes
• Higher ratings of having received sufficient information about diagnosis and treatment were associated with a lower likelihood of a good treatment response.

**HOW THIS STUDY MIGHT AFFECT RESEARCH, PRACTICE, OR POLICY**

• Early intervention is crucial for optimizing treatment results.
• Patient information should be individually tailored to enhance motivation and foster hope.
• Involving patient research partners in developing the content and delivery of information and self-management strategies is essential to ensure relevance and effectiveness for the target population.

Alt-text: Unlabelled box dummy alt text

## INTRODUCTION

Hand osteoarthritis (HOA) is a rheumatic disease that affects both the hand joints and surrounding tissues. There is an estimated 40% lifetime risk of developing HOA, with women being at higher risk [1,2]. Accompanying symptoms from HOA include pain, stiffness, and impaired function. This may negatively impact work participation and health-related quality of life and impose substantial socioeconomic burdens [3,4]. Despite its high prevalence, no cure exists, highlighting the need for effective management strategies [5].

There is a general consensus across treatment recommendations that information, hand exercises, and guidance in use of ergonomic working methods and assistive devices should be provided as first-line treatment, often supplemented by provision of thumb orthoses and short-term use of pain-relieving medication [6,7]. Studies have shown that these interventions are effective in reducing pain and enhancing hand function in the short term; however, their long-term efficacy remains less evident [8]. Surgery should be considered if all other interventions have failed to sufficiently relieve pain and improve function [5].

Improving care models for HOA requires a comprehensive understanding of the factors that are associated with favorable treatment outcomes at a given timepoint. Despite their importance, such factors for HOA remain underexplored. Therefore, the aim of this study was to explore patient- and healthcare factors associated with treatment response six months after having received recommended treatment for HOA in specialist healthcare.

## MATERIALS AND METHODS

### Study design

The current study extends the data from a multicentre noninferiority randomised controlled trial (RCT), as predefined in the original RCT protocol and the statistical analysis plan [9]. The main aims of the RCT were to assess the noninferiority and cost-effectiveness of occupational therapist-led care for HOA compared with rheumatologist-led care. Data were collected at Martina Hansen Hospital and Diakonhjemmet Hospital, both located in the south-eastern part of Norway. The Standard Protocol Items: Recommendations for Interventional Trials (SPIRIT guidelines) were used, and the protocol was registered at clinicaltrials.gov on April 5, 2017 (NCT03102788).

### Participants

Participants were considered eligible for the RCT if they had been diagnosed with HOA by a general practitioner and were referred to either Martina Hansen Hospital or Diakonhjemmet Hospital for further evaluation. Inclusion criteria were age >18 years and having proficiency in Norwegian language. Exclusion criteria were having psoriasis, severe psychiatric disorder, cognitive impairment, or possible inflammatory rheumatic disease (erythrocyte sedimentation rate >40 mm/h/C-reactive protein >20 mg/L). Exclusion criteria were assessed based on information from medical records, results from blood test, and the initial patient contact.

The recruitment procedure is described in detail elsewhere [9]. In short, patients referred to a specialist consultation for HOA were identified from the hospital referral list. Potentially eligible patients were sent written information about the study and contacted the subsequent week for initial screening. Patients who met the eligibility criteria and consented verbally were asked to sign a consent form and complete a questionnaire to bring to their hospital appointment. Patients who provided written consent were enrolled in the study upon arrival at the hospital. On the same day, a research assistant (occupational therapist or physiotherapist) not involved in the recruitment or treatment, performed the baseline assessment of the participants. Data were collected between September 2017 and November 2020 from clinical assessments, questionnaires, medical records, and radiographs.

### Interventions

The interventions followed the European Alliance of Associations for Rheumatology (EULAR) recommendations for the management of HOA [5]. Depending on group allocation, participants underwent a 45 minutes consultation that included clinical examination by an experienced rheumatologist or occupational therapist, providing oral and written information about HOA and treatment options including pharmacological treatment and orthoses. Treatment was individualised through dialogue with the patient, assessment of the Measure of Activity Performance of the Hand (MAP-Hand) which contained standardised and patient-defined activities, and relevant clinical tests, such as the Jamar grip strength measurement. The intervention was subsequently tailored to the patient’s symptoms, functional limitations, goals and clinical findings, and could include education about HOA, guidance in ergonomic work methods, use of assistive devices, and/or hand exercises, as well as provision of hand orthoses. Occupational therapists complemented their verbal guidance by giving instructions on hand exercises, as well as supplying orthoses, if needed. They also distributed a booklet containing information about HOA, pharmacological treatment, ergonomic principles, assistive devices, orthoses, and hand exercises (Supplementary Appendix). Rheumatologists provided advice on pharmacological treatment and administered intra-articular steroid injections to participants with inflammation in interphalangeal or first carpometacarpal (carpometacarpal joint 1 [CMC1]) joint(s) when appropriate. The treatment was mostly provided in one visit, although some patients required two visits. Both occupational therapists and rheumatologists could refer participants to each other or other services, such as, for example, an orthopaedic surgeon. Regardless of allocation, some patients also attended a 1-day group education program on OA and/or a hand exercise group led by an occupational therapist. Follow-ups were conducted at 6 and 12 months. The main conclusion of the original RCT was that occupational therapist–led care for HOA was noninferior to rheumatologist-led care with respect to efficacy, safety, and patient satisfaction, while also demonstrating greater cost-effectiveness [10].

### Outcomes and measures

As in the original RCT, the primary outcome of the current study was response to treatment measured by the OMERACT-OARSI responder criteria 6 months after the initial consultation [11]. This is a composite index that classifies participants as either responders (yes) or nonresponders (no), based on predefined absolute or relative changes after treatment within 3 domains: pain, function, and patient’s global assessment of disease activity.

Other data collected at baseline included patient-reported information about age, sex (biological and physiological characteristics used to classify individuals as male or female), work and marital status, comorbidities, height and weight, years with symptoms from finger joints, and use of analgesics. Comorbidities were measured using a standardised questionnaire where patients were asked to indicate if they had one or more of the following conditions: lung disease, allergy, low back pain, stroke, neurologic disease, cancer, diabetes, kidney or liver disease, metabolic disease, psychological disease, blood disease, and gastrointestinal disease. A skilled assessor (occupational therapist or physiotherapist) examined all joints in both hands to determine the presence of pressure pain and bony enlargement. A score of 1 indicated presence and 0 indicated absence. Pain scores ranged from 0 to 14 for each hand (CMC1, metacarpophalangeal, and the interphalangeal joints of the second through fifth fingers). Bony enlargement scores ranged from 0 to 9 (all interphalangeal joints). Radiographic HOA severity of the CMC1 joint was assessed using a modified Kellgren-Lawrence (KG) scale, with 0 indicating no osteoarthritis and 4 reflecting severe osteoarthritis [12]. We categorised CMC1-OA as having a KG-score of grade ≥2 in at least 1 CMC1 joint. Grip strength was measured using the JAMAR hydraulic hand dynamometer, adhering to established protocols [13]. Additionally, patients reported their level of disease activity, stiffness, and hand pain at rest on 0 to 10 numerical rating scales (NRS), where higher scores indicate greater symptom severity. The MAP-Hand was used to assess hand function [14,15]. This is a patient-reported 18-item instrument, with scores ranging from 1 (= no difficulty) to 4 (= unable to do). A mean index score based on a minimum of 15 completed items was calculated. The general health item from the EuroQol 5-Dimension 5-Level Visual Analogue Scale was used to measure general health on a scale ranging from 0 to 100, where higher scores reflect better health [16].

Patients’ experience with received healthcare was evaluated by the PasOpp questionnaire, which participants completed at the 6‑month follow‑up visit. PasOpp is a Norwegian survey instrument consisting of 12 items that measure patients’ experiences with health services [17]. Each item was analysed separately, as the PasOpp is not designed to generate a mean or total sum score. Items 1 to 10 (see Table 1) were rated on 5-point Likert scales, with items 1 to 9 ranging from ‘not at all’ to a ‘very large extent’ and item 10 (‘suspected mistreatment’) from ‘no extent’ to ‘a high extent’. Item 11 (waiting time for hospital appointment) was assessed using a 4-point scale ranging from ‘not at all’ to ‘all too long’, whereas item 12 (‘perceived benefit of treatment’) was rated on a 5-point Likert scale where a score of 5 indicates ‘very high benefit’.

**Table 1:** Patients experiences with healthcare measured by the PasOpp questionnaire

	Total sample (N = 340)
	Median (IQR)
1. Clear communication from providers (1–5, 5 = very large extent)	5.0 (4.0-5.0)
2. Confidence in healthcare providers competence (1–5, 5 = very large extent)	5.0 (4.0-5.0)
3. Sufficient information, procedures/examinations (1–5, 5 = very large extent)	4.0 (4.0-5.0)
4. Sufficient information about diagnosis/treatment (1–5, 5 = very large extent)	4.0 (4.0-5.0)
5. Treatment tailored to situation (1–5, 5 = very large extent)	4.0 (4.0-5.0)
6. Involvement in treatment decisions (1–5, 5 = very large extent)	4.0 (3.0-5.0)
7. Institutions work well-organised (1–5, 5 = very large extent)	5.0 (4.0-5.0)
8. Equipment in good condition (1–5, 5 = very large extent)	5.0 (4.0-5.0)
9. Satisfaction with treatment (1–5, 5 = very high extent)	4.0 (4.0-5.0)
10. Suspected mistreatment (1–5, 1 = not at all)	1.0 (1.0-1.0)
11. Waiting time (1–4, 1 = not at all)	2.0 (1.0-2.0)
12. Perceived benefit of treatment (1–5, 5 = very high benefit)	3.0 (3.0-4.0)

PasOpp: Norwegian national survey instrument used to measure patients' experiences with healthcare services.

### Patient and public involvement

Two patient research partners with experience from living with HOA were part of the research group. They contributed to the study design, development of research questions, and strategies for optimising the recruitment process. Their contributions were reimbursed according to Norwegian standard rates. One member retired during the study period. The other (TS) remained actively involved throughout all phases of the trial and is included as a coauthor.

### Ethical considerations

All participants received what was considered standard recommended treatment following procedures described in the study protocol [9]. Participants suspected of having inflammatory rheumatic disease were excluded from the study and referred to a rheumatologist. The study adhered to the principles outlined in the Declaration of Helsinki and received approval from the Norwegian Regional Committee for Medical Research Ethics (2017/742/REK Sør-Øst A). The data protection officer at Diakonhjemmet Hospital verified that the study fulfilled the General Data Protection Regulation requirements (2020/00184).

### Statistical methods

Sample size was based on the requirements for the original RCT [9]. Descriptive statistics were utilised to summarise the sample’s characteristics, with the mean and SD or median and IQR being reported for continuous variables, and frequencies and proportions for categorical variables. Because the drop-out rate was low (≤10%), we did not impute missing data.

We conducted 2 regression analyses to assess factors associated with being an OMERACT-OARSI responder (yes/no). In both analyses, multivariable logistic regression was applied, first including all variables (initial full model) and then with backward deletion, removing the variable with the highest *P* value until only significant variables and covariates remained (final model). Age, sex, and group allocation were included as covariates. In the first analysis, patient-related variables were included as independent variables, while in the second analysis, experiences with healthcare were the independent variables. Before conducting the regression analyses involving patient-related variables, grip strength was consolidated into a single measure by averaging the values from both hands. With regard to healthcare variables, responses from items 10 and 11 in the PasOpp were reversed to ensure a coherent interpretation of results. Further, multicollinearity was evaluated using Pearson correlation, variance inflation factor (VIF), and tolerance statistics, with thresholds set at Pearson *r* < 0.7, VIF > 5, and tolerance <0.2 to identify potential issues. The logistic regression analyses were done on complete cases. Results are reported as odds ratio (OR) with 95% CIs and *P* values.

## RESULTS

A total of 512 eligible patients were invited to participate. Of these, 110 declined to participate, leaving 402 scheduled for baseline assessment. Twelve participants withdrew before baseline assessment due to illness, family reasons, relocations, or unspecified reasons; thus, 390 completed the baseline assessment. Furthermore, 18 participants thereafter withdrew due to illness, family reasons, relocation or other reasons, resulting in 374 participants receiving treatment for HOA. One participant died and 33 withdrew before the 6-month follow-up; thus, data from 340 participants were analysed (Fig). When comparing the baseline values of those who attended 6 months follow-up with those who did not, those not attending reported significantly higher disease activity, more reduced hand function, higher pain levels at rest, higher numbers of painful joints, and more pain during activity in their right hand (data not shown).

**Figure:**
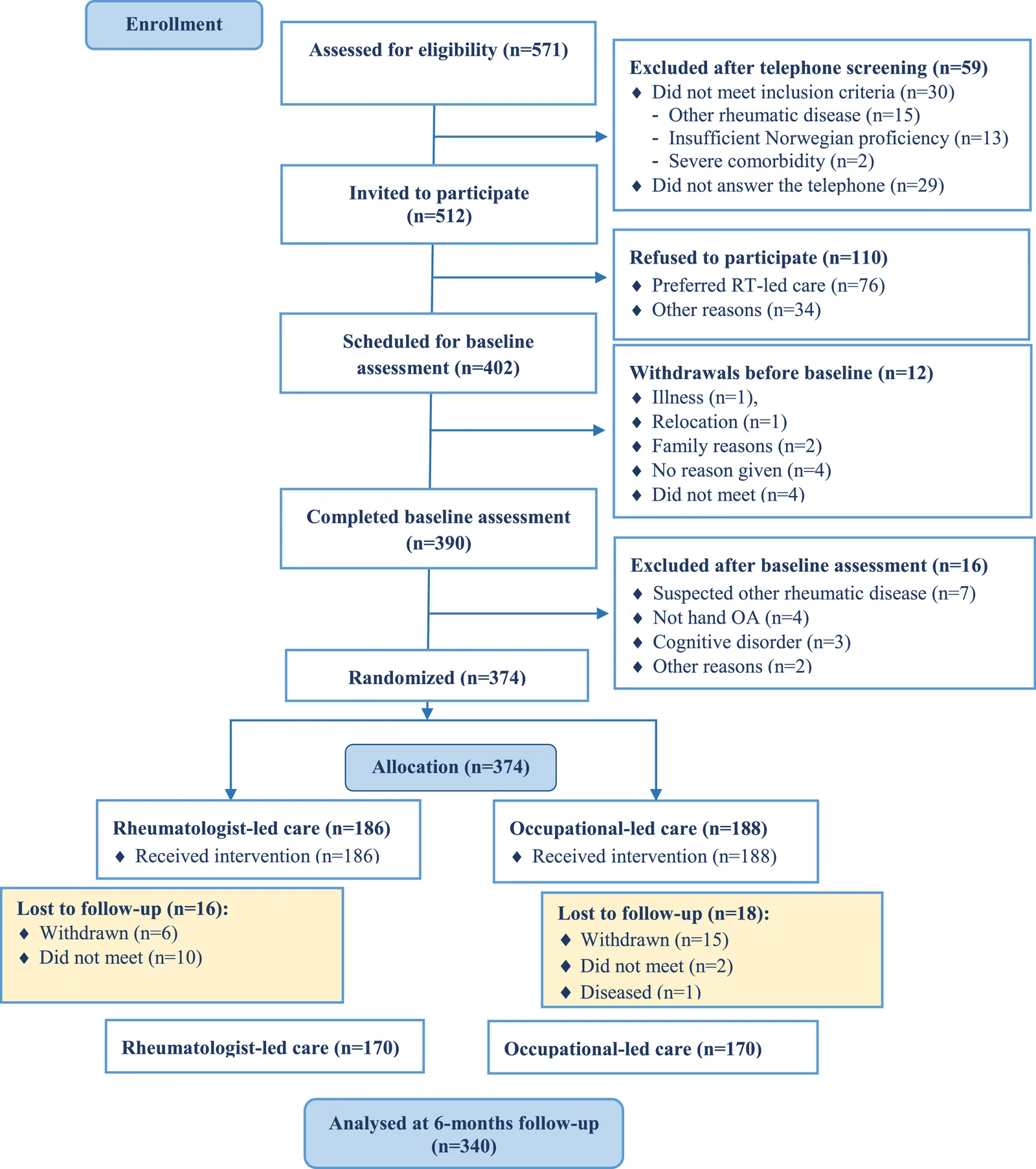
Flow diagram of the original randomised controlled trial. OT, occupational therapist; RT, rheumatologist.

As shown in Table 2, the average age of study participants was 63.6 years, with 80.7% being female and 70.3% living with a partner. They had been living with HOA symptoms for a median of 5.5 years, and 75.1% had coexisting comorbidities. At baseline, participants reported moderate levels of disease activity, hand pain and activity performance, and perceived their general health to be moderately good. More than half of the participants had a KL score of ≥2 in the CMC1 joint, indicating moderate-to-severe radiographic HOA. Almost half used analgesics to relieve pain.

**Table 2:** Baseline characteristics of study patients

	Total sample (n = 374)
Age, y, mean ± SD	63.6 ± 10.0
Female sex, n (%)	302 (80.7)
Education, 12 y or more, n (%)	218 (58.3)
Marital status, partnered, n (%)	263 (70.3)
Employed, n (%)	219 (58.6)
BMI (kg/m^2^), mean ± SD	25.6 ± 4.5
One or more comorbidities, n (%)	281 (75.1)
Symptom duration, y, median (IQR)	5.5 (2.0-13.0)
Analgesic use, n (%)	185 (49.5)
Disease activity (NRS, range: 0-10), mean ± SD	5.0 ± 2.1
Hand pain at rest (NRS, range: 0-10), median (IQR)	5.0 (3.0-7.0)
Stiffness hand (NRS 0-10), mean ± SD	4.8± 2.4
Hand function (MAP-Hand, range: 1-4), mean ± SD	1.9 ± 0.5
OA severity in right CMC1 (KL grade ≥ 2), n (%)	174 (52.4)
OA severity in left CMC1 (KL grade ≥ 2), n (%)	189 (59.1)
No. noduli, both hands (range: 0-18), median (IQR)	5.0 (2.0-10.0)
No. painful joints, both hands (range: 0-28), median (IQR)	11.0 (6.0-17.0)
Grip strength in kg, right hand, mean ± SD	20.4 ± 10.0
Grip strength in kg, left hand, mean ± SD	19.1 ± 9.8
General health (EQ-5D-5L, VAS, range: 0-100), mean ± SD	64.9 ± 20.3

CMC1, first carpometacarpal joint (base of the thumb); EQ-5D-5L VAS, EuroQol 5-Dimension 5-Level Visual Analogue Scale, FIOHA, Functional Index for Hand Osteoarthritis; KL Grade, Kellgren-Lawrence grade; MAP-Hand, Measure of Activity Performance of the Hand; No, number; NRS, Numerical Rating Scale; OA, osteoarthritis.

After 6 months, 48 patients from each treatment group were identified as OMERACT-OARSI responders, resulting in a total of 96 (28%) responders in the total sample [10]. When looking at patient-related factors associated with treatment response, we found that participants with less advanced radiographic CMC1-OA (KL grade <2) had nearly double the odds for achieving good treatment response compared to those with more severe osteoarthritis (OR: 1.95, 95% CI: 1.07, 3.55). Furthermore, for each 1-point worsening in hand pain score on the NRS scale, the odds for being a responder increased by 18% (OR: 1.18, 95% CI: 1.05, 1.33) (Table 3).

**Table 3:** Patient factors associated with responding to treatment at 6 mo in patients with hand osteoarthritis (OA) (n = 340)

	Initial full model			Final model (n = 281)		
Factors	OR	95% CI	*P* value	OR	95% CI	*P* value
Age (y)	1.00	0.97-1.04	.92	1.00	0.98-1.03	.79
Male sex	1			1		
Female sex	0.71	0.29-1.72	.45	0.67	0.35-1.26	.21
Rheumatologist-led care model group 1	1			1		
Occupational therapist care model group 2	1.23	0.72-2.10	.44	1.22	0.72-2.07	.45
CMC1 OA (K-L grade ≥2)	1			1		
CMC1 OA (K-L grade <2)	**2.05**	**1.11-3.75**	**.02**	**1.95**	**1.07-3.55**	**.03**
Hand pain at rest (NRS)	**1.28**	**1.05-1.55**	**.01**	**1.18**	**1.05-1.33**	**.01**
Stiffness (NRS)	1.06	0.90-1.23	.51			
Hand function (MAP-hand)	0.67	0.31-1.47	.32			
Grip strength, mean of both hands (JAMAR in kg)	1.00	0.98-1.02	.97			
General health (EQ-5D-5L VAS)	1.01	1.00-1.03	.14			

CMC1, carpometacarpal joint 1; EQ-5D-5L VAS, EuroQol 5-Dimension 5-Level Visual Analogue Scale; JAMAR, dynamometer; K-L grade, Kellgren-Lawrence grade; MAP-Hand, Measure of Activity Performance of the Hand; NRS, Numerical Rating Scale; OA, osteoarthritis.

Bold values in Table 3 indicate which independent factors were significantly associated with the dependent variable.

Participants consistently reported positive experiences with the healthcare received at the outpatient clinic, with median scores of 4 or 5 in the first 9 items. Further, the median score for suspected mistreatment was 1 = ‘not at all’, the median score for waiting time was 2 = ‘yes, but not long’, whereas the median score for perceived treatment benefit was 3, indicating a moderate benefit (Table 1).

The results further showed that reporting high and positive scores of ‘trust in healthcare providers competence’ and ‘perceived treatment benefit’ were positively associated with good treatment response, with ORs of 2.09 (95% CI: 1.10, 3.96) and 1.74 (95% CI: 1.28, 2.36), respectively (Table 4). Conversely, high scores of ‘sufficient information about diagnosis and treatment’ significantly decreased the odds for good treatment response, with an OR of 0.63 (95% CI: 0.42, 0.96) (Table 4).

**Table 4:** Healthcare factors associated with responding to treatment at 6 mo in patients with hand osteoarthritis

	Initial full model			Final model (n = 333)		
Factors	OR	95% CI	*P* value	OR	95% CI	*P* value
Age (y)	1.00	0.97-1.03	.78	0.99	0.96-1.01	.28
Male sex	1			1		
Female sex	0.66	0.31-1.39	.27	0.67	0.37-1.23	.20
Rheumatologist-led care model group 1	1			1		
Occupational therapist care model group 2	0.88	0.47-1.65	.69	1.02	0.61-1.70	.95
Patients experience with healthcare						
1. Clear communication from providers	0.98	0.42-2.29	.97			
2. Trusted healthcare providers competence	2.29	0.89-5.88	.09	**2.09**	**1.10-3.96**	**.02**
3. Sufficient information, procedures/examinations	1.34	0.66-2.74	.42			
4. Sufficient information, diagnosis/treatment	**0.49**	**0.26-0.91**	**.02**	**0.63**	**0.42-0.96**	**.03**
5. Treatment tailored to situation	**0.55**	**0.31-0.99**	**.045**			
6. Involvement in treatment decisions	0.88	0.57-1.36	.55			
7. Institutions work well-organised	0.60	0.31-1.17	.13			
8. Equipment in good condition	1.39	0.65-3.00	.40			
9. Satisfaction with treatment	1.87	0.92-3.80	.08			
10. Suspected mistreatment	1.03	0.70-1.52	.87			
11. Waiting time	0.83	0.49-1.42	.50			
12. Perceived treatment benefit	**2.01**	**1.27-3.18**	**.003**	**1.74**	**1.28-2.36**	**<.001**

Bold values in Table 4 indicate which independent factors were significantly associated with the dependent variable.

## DISCUSSION

To our knowledge, there is a lack of studies examining how patient characteristics and their experiences with healthcare delivery impact treatment outcomes in HOA. Our study addresses this gap by exploring factors associated with treatment response 6 months posttreatment for HOA. Regarding patient-related factors, the results showed that having milder OA in the CMC1 joint (K-L grade <2) almost doubled the likelihood of being classified as a responder to the treatment received. Furthermore, higher pain levels were also associated with a positive treatment response, with an 18% increase in likelihood for each point on the NRS scale.

In our study, the median years with HOA symptoms was 5.5 years, and more than half had severe HOA in their CMC1 joint. The finding that milder HOA is associated with good treatment response suggests that individuals with HOA should receive treatment for their condition as soon as possible after the onset of symptoms, before the disease causes too much joint destruction. One challenge to this is the well-documented discordance between symptoms and radiographic changes, which complicates early diagnosis [18]. Another barrier is that healthcare professionals often inform patients that HOA is a part of normal aging and that nothing can be done to treat it, thereby limiting patients’ access to treatment [19,20]. Furthermore, research shows that although current strategies recommend that HOA should mainly be treated in primary care, the available services there are very limited, with other patients being prioritised at the expense of those with HOA [21].

A promising strategy to overcome these barriers is the use of mobile applications (apps). To date, two apps have been developed for HOA. One is the CareHand app, which demonstrated superior results compared to usual care in improving hand function and reducing pain [22]. The other is the Happy Hands app, which was evaluated in a feasibility study with 71 patients with HOA. Participants found the app to be motivating and easy to use and reported improvements in pain, stiffness, grip strength, and activity performance after using the app for three months [23]. Given that apps can be downloaded regardless of where people live and their contact with the healthcare system, they may be an effective way to bypass previously described barriers and ensure optimal utilisation of healthcare resources.

Pain is the cardinal symptom of OA, and higher pain levels are associated with central pain sensitisation, sleep problems, and reduced function and health-related quality of life [4,[24], [25], [26]]. Consequently, there is consensus across all treatment guidelines that individuals with HOA should be offered pain-relieving treatment [6,7].

In a recent review of treatments for HOA, meta-analyses demonstrated that thumb orthoses, hand exercises, and nonsteroid anti-inflammatory drugs were effective in reducing pain [8]. In line with these findings, international guidelines recommend that pain should be routinely assessed during consultations, all patients should receive guidance on hand exercises, individuals with thumb base pain should be offered orthoses for long-term use to alleviate pain, and pharmacological treatments should be used short-term to relieve pain and support therapeutic exercise [6,7].

In our study, individuals with higher levels of pain were more likely to benefit from treatment for their HOA. This may reflect the nature of the interventions they received, including pharmacological treatment, use of orthoses, exercise programs, assistive devices, and ergonomic strategies incorporated into daily activities. However, this finding may also be partly attributable to regression to the mean, as patients often seek care when their symptoms are at their worst, and symptom severity in OA is known to fluctuate over time [27]. Additionally, patients reporting more severe pain may have been more likely to express their discomfort clearly and, consequently, more frequently received recommended pain-relieving interventions.

Moving on to healthcare factors, positive ratings of ‘trust in healthcare providers competence’ and ‘perceived treatment benefit’ were positively associated with good treatment response. This finding aligns with results from a qualitative study in which participants with HOA highlighted the importance of trust in healthcare professionals’ expertise and communication skills as a foundation for effective interactions during consultations [20]. All clinicians in our study had up-to-date knowledge and extensive experience in treating patients with HOA [10]. This is reflected in the participant’s high trust in their expertise, and may also lead to greater adherence to the advice provided by the practitioners, which in turn results in a good treatment response. To enhance clinician’s competence, they should be provided with opportunities for education and training in effective communication and examination methods, as well as to stay updated on research in the field.

The positive association with perceived treatment benefits also indicates that patients’ self-assessment of improvement aligns with the aspects included in the OMERACT-OARSI responder calculation, and is in line with previous studies concluding that patient-reported outcome measures are sensitive to capture changes in their health and well-being [28,29].

There is a general agreement across treatment guidelines that management of HOA should be individualised according to joint localisation, severity and comorbidities and include information on the nature, course, and treatment of the disease [6,7]. It is therefore somewhat surprising that high ratings of having received sufficient information about diagnosis and treatment were negatively associated with positive treatment response. One reason may be that healthcare providers offer more detailed—and potentially frightening—information about the disease and its progression than patient’s desire. Combined with learning that there is no cure for the disease, this may lead to feelings of resignation and loose of hope for improvement. This hypothesis is supported by findings in a study on the development of a self-management app for people with HOA, where patient research partners giving input to the app design were very clear that information should be motivating and encouraging, and that “worst-case scenarios” and frightening illustrations should be avoided [23]. Similarly, results from an RCT showed that removing pathoanatomical content from educational materials reduced beliefs that exercise is harmful and improved disease knowledge in patients with knee OA [30]. How patient information should be tailored to promote hope and motivation for self-management should therefore be explored further in future studies. Involving patient research partners in developing both the content and format of such information is essential to ensure that it effectively reaches the target group [31].

### Limitations of the study

A limitation of our study is that we used data from an RCT and treated it as a prospective cohort study. However, the results from the RCT did not show any differences in response rates between the two groups, and we controlled for group allocation in our analyses [10]. Another limitation is that participants who dropped out of the study had higher disease activity, more pain at rest, more painful finger joints, and poorer hand function than those included in the analyses. However, if they had been included, the association between pain at rest and treatment response might potentially have been even stronger. Also, the study did not include a placebo group. Given the high placebo response reported in this population, the observed effect may partly reflect placebo [32,33]. As the study had an exploratory aim, the use of *P* value–based backward elimination is a recommended approach in such contexts. Nonetheless, it may increase the risk of overfitting and the exclusion of clinically relevant variables. Although the relatively large sample size may have mitigated these risks, the findings should be interpreted as exploratory rather than causal or predictive [34,35]. Lastly, in 2024, EULAR’s classification of HOA was published, categorising it into overall HOA, interphalangeal OA, and thumb base OA [36]. Unfortunately, we do not have the data in our study to apply this classification and instead used the KL score of the CMC1-joint to explore whether radiographic severity was associated with responder status. To better identify which patients would benefit most from different interventions, future studies should investigate whether treatment response varies between the 3 types of HOA.

## CONCLUSION

Our study addresses the gap in research regarding how patient and healthcare factors impact treatment outcomes for HOA. The results show that less severe HOA and higher pain levels significantly increased the likelihood of a positive treatment response. Early intervention is therefore crucial.

High trust in healthcare providers' competence and high treatment benefit were also associated with better outcomes, whereas high ratings of having received sufficient information about diagnosis and treatment reduced the likelihood of a good treatment response. These findings underscore the importance of tailoring patient information to foster motivation and hope. Future studies should investigate how best to design and deliver patient information to optimise treatment outcomes.

## CRediT authorship contribution statement

**Unni Olsen:** Writing – review & editing, Writing – original draft, Visualization, Methodology, Investigation, Formal analysis. **Anne Therese Tveter:** Writing – review & editing, Writing – original draft, Visualization, Validation, Supervision, Software, Resources, Methodology, Investigation, Formal analysis, Conceptualization. **Kjetil Bergsmark:** Writing – review & editing, Validation, Resources, Data curation, Conceptualization. **Barbara S. Christensen:** Writing – review & editing, Validation, Data curation, Conceptualization. **Ida K. Haugen:** Writing – review & editing, Methodology, Investigation, Formal analysis, Conceptualization. **Toril Hennig:** Writing – review & editing, Validation, Resources, Project administration, Funding acquisition, Data curation, Conceptualization. **Merete Hermann-Eriksen:** Writing – review & editing, Validation, Project administration, Data curation, Conceptualization. **Åshild Hove:** Writing – review & editing, Validation, Data curation, Conceptualization. **Trine A. Sjøvold:** Writing – review & editing, Validation, Methodology, Conceptualization. **Ingvild Kjeken:** Writing – review & editing, Writing – original draft, Visualization, Validation, Supervision, Resources, Project administration, Methodology, Investigation, Funding acquisition, Formal analysis, Data curation, Conceptualization.

## Competing interest

IKH has received consulting fees from Novartis, GSK, and Grünenthal, and is a member of the OARSI executive committee. All other authors declare no competing interests.

## Appendix Supplementary materials

Image, application 1

## Footnotes

Supplementary material associated with this article can be found in the online version at doi:10.1016/j.ero.2026.100204.
