# Supplementary material for: Patient and healthcare factors associated with treatment response in patients with hand osteoarthritis: secondary analyses from a randomised controlled, noninferiority trial
Source: EULAR Rheumatol Open. 2026 Jun 26;2(3):100204. doi: 10.1016/j.ero.2026.100204 (PMC13650361; doi:10.1016/j.ero.2026.100204)

Information for people with hand osteoarthritis

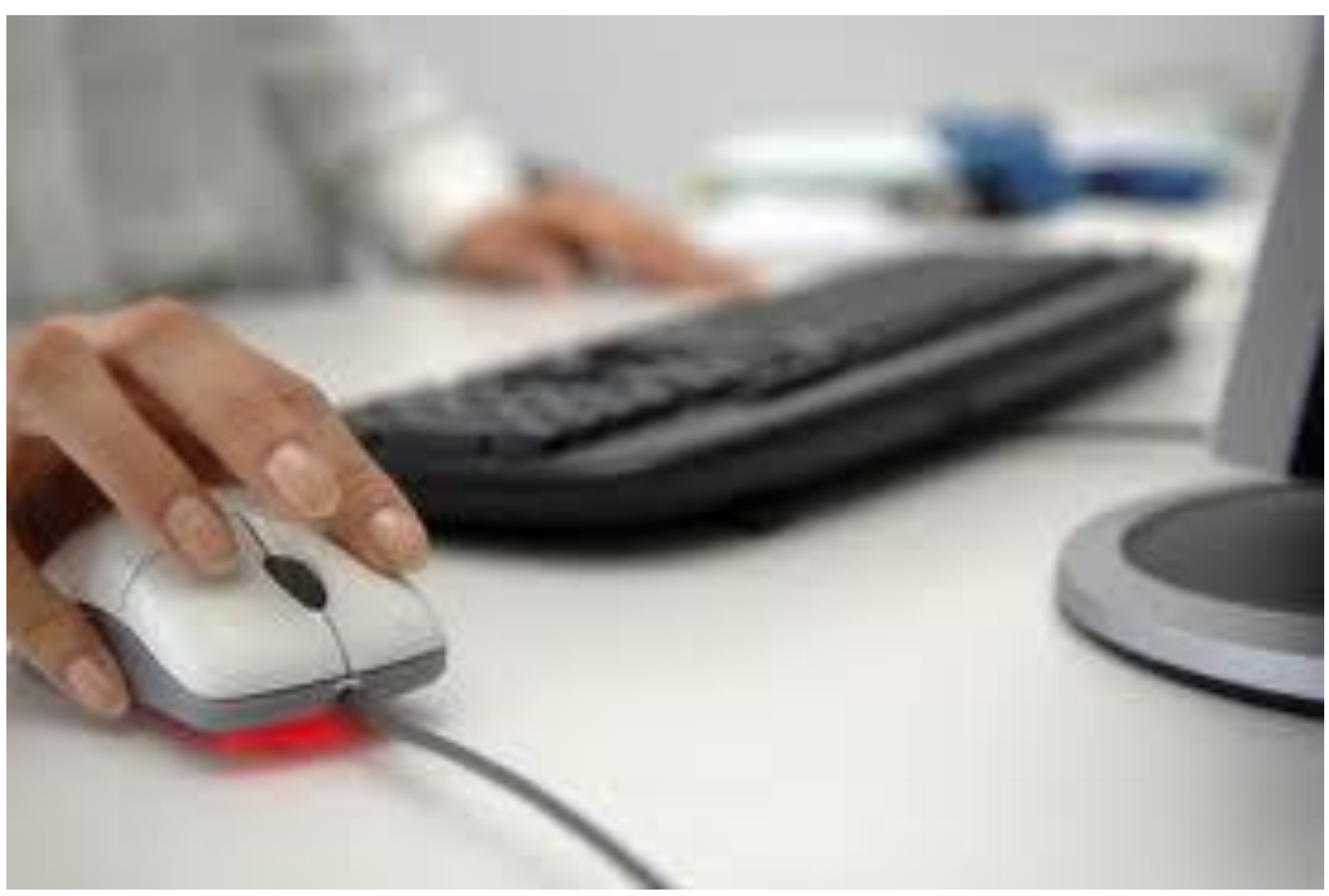

Authors: Åshild Hove, Madeleine S-Strøm, Helle Haveraaen, Cathrine Østvik, Merete Hermann-Eriksen, Aud Rønningen and Ingvild Kjeken.

August 2017.

In this booklet, you will find information about hand osteoarthritis and pharmacological treatment, advice on the use of ergonomic working methods, assistive devices and orthoses, and an exercise program.

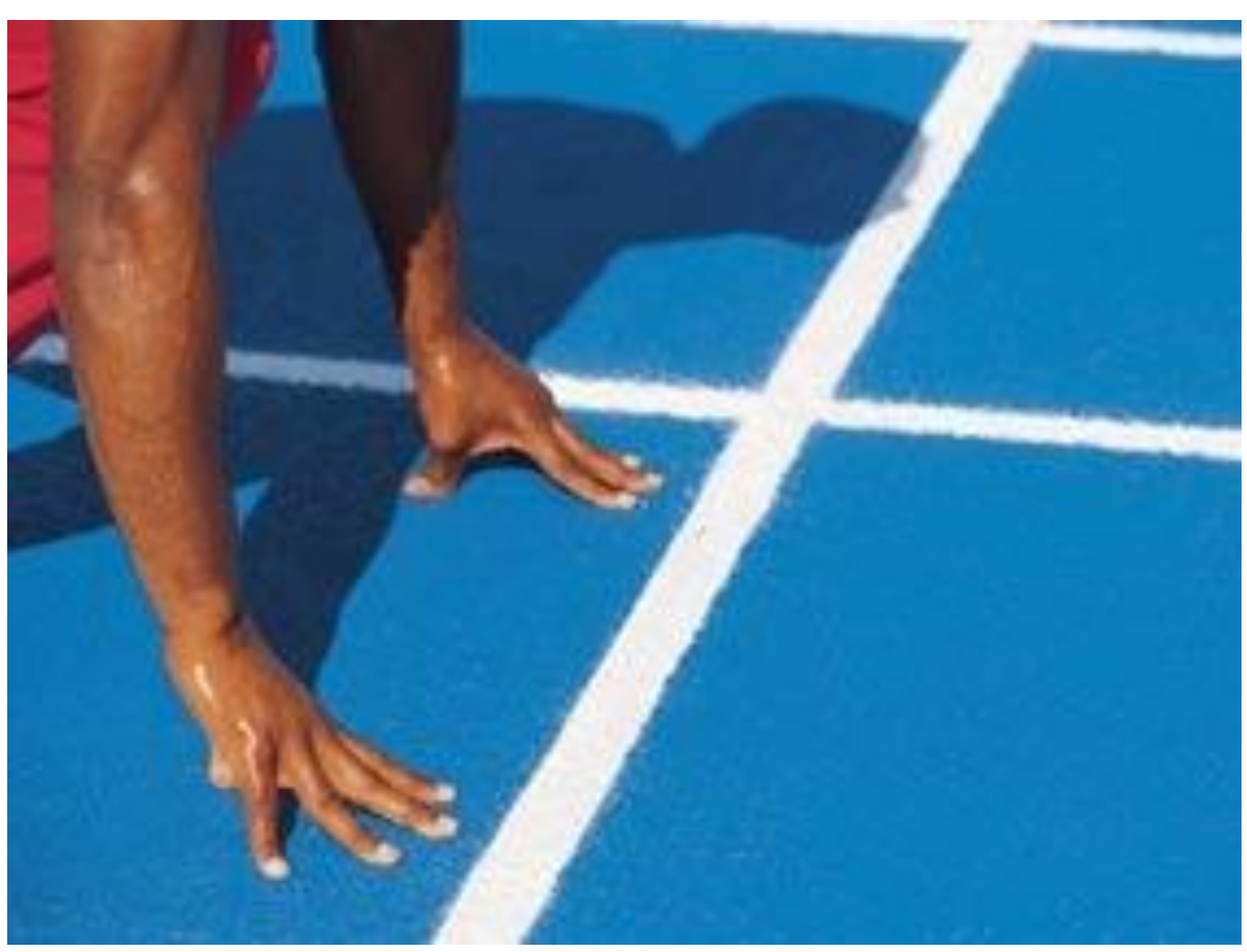

OSTEOARTHRITIS

Osteoarthritis is the most common of the rheumatic diseases. It is a joint disorder that, over time, leads to a gradual deterioration of the cartilage within the joint. In the past, the term “wear-and-tear arthritis” was used, but this is misleading, as it may suggest that the cartilage or joint has been worn out due to use. Today, osteoarthritis is more often regarded as a disease in which the normal balance between the breakdown and formation of joint cartilage is disrupted.

The condition affects more women than men, and its prevalence increases with age. Osteoarthritis most commonly occurs in the knees, hips, hands, and spine. Typical symptoms include pain and stiffness in the joints. Some individuals may develop joint deformities, and some may also experience increased fatigue.

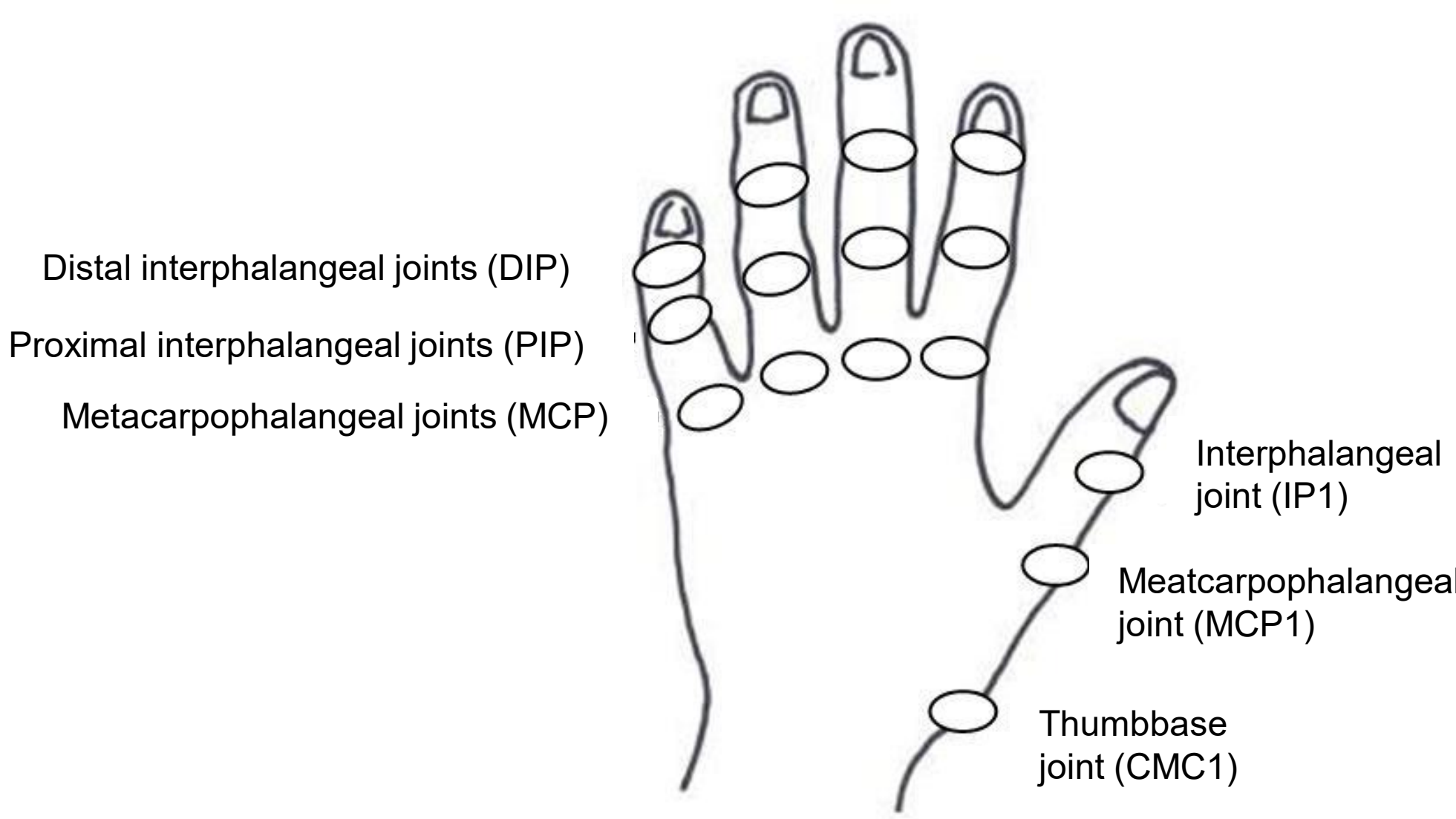

HAND OSTEOARTHRITIS

The diagnosis of hand osteoarthritis is primarily based on a clinical examination by a physician and can be confirmed using X-ray and ultrasound. In hand osteoarthritis, the most commonly affected joints are the distal and proximal interphalangeal joints as well as the thumb base joint. Visible bony enlargements are often present, appearing as nodules at the distal joints, a general thickening around the middle joints, or a more prominent appearance of the thumb base joint.

Symptoms often begin with diffuse pain in the thumb or in the middle and distal joints of the fingers. Grip strength becomes reduced, and activities such as wringing out cloths or opening jam jars may become difficult. Tasks requiring fine motor skills and precision may also become more challenging. Stiffness is usually most pronounced in the morning and after periods of inactivity and often improves with movement. Many people find that their symptoms worsen in cold weather and during changes in weather conditions.

At times, the joints may become inflamed, with increased warmth, tenderness, and redness. This inflammation is usually mild and short-lived. Occasionally, episodes of acute pain and swelling may occur. Osteoarthritis at the base of the thumb often causes pain throughout the entire thumb, which may also radiate into the wrist. Osteoarthritis in the base joint may also lead to development of deformities in other thumb joints.

Movement and use of the body improve the nourishment of the cartilage and help strengthen the joints. It is therefore now generally agreed that normal use and loading of the hands do not accelerate the disease; rather, they help maintain mobility and hand strength.

Symptoms of hand osteoarthritis may decrease over time and are rarely disabling.

PHARMACOLOGICAL TREATMENT OF HAND OSTEOARTHRITIS

There are currently no medications that slow the progression of osteoarthritis. The aim of drug treatment is therefore to reduce pain and stiffness and thereby improve function.

The most common groups of medications used in osteoarthritis are:

- Analgesics (pain relief): Paracetamol is often the first choice. Stronger painkillers may sometimes be necessary.
- Anti-inflammatory drugs (NSAIDs): These medications do not contain corticosteroids. Examples are ibuprofen (Ibux), naproxen, and diclofenac.
- Injection therapy: In cases of significant swelling and pain in individual joints, corticosteroid injections may be appropriate.

Symptoms of osteoarthritis may vary over time. It is therefore important to adjust pharmacological treatment accordingly. Medications should preferably be used only when needed.

ERGONOMIC WORKING METHODS AND USE OF ASSISTIVE DEVICES

People with osteoarthritis in the thumb may experience pain and reduced grip function, which can affect performance of daily activities. Most people carry out activities automatically without thinking about how their joints are being used. When living with hand osteoarthritis, alternative working techniques and assistive devices can make it easier to perform valued activities with as little pain as possible.

It is also important to balance activity and rest, take breaks, vary working methods and avoid repetitive strain. Below are some examples of good working techniques and assistive devices.

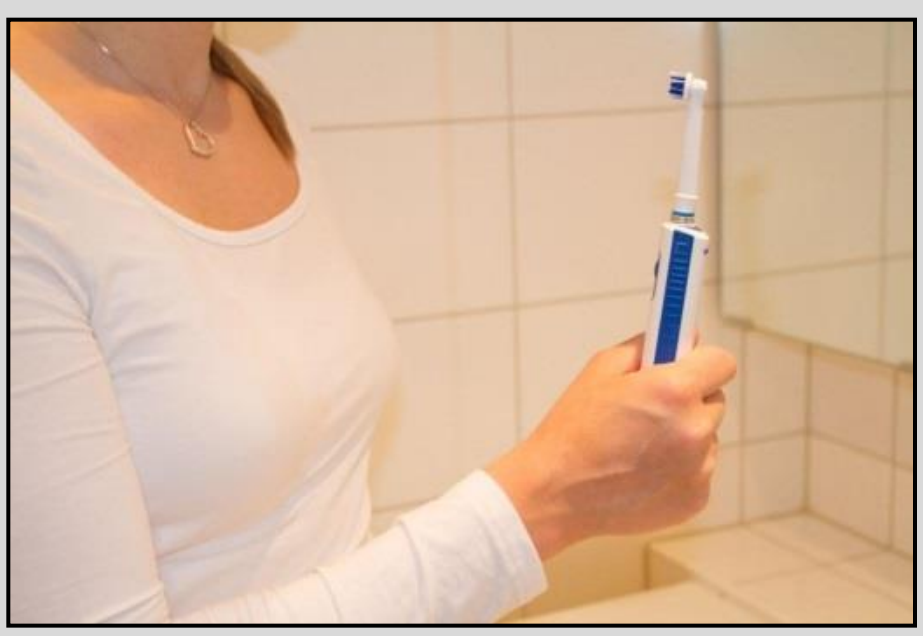

Using larger grips helps reduce stress on your joints.

.

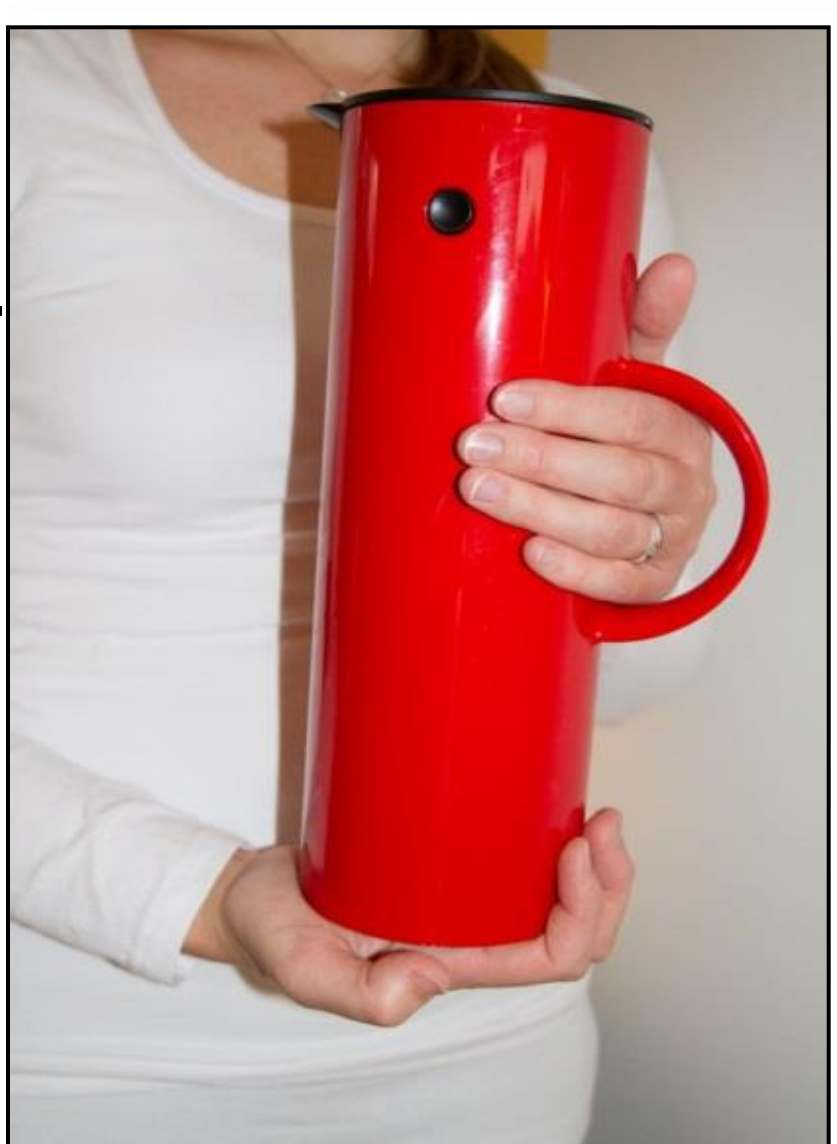

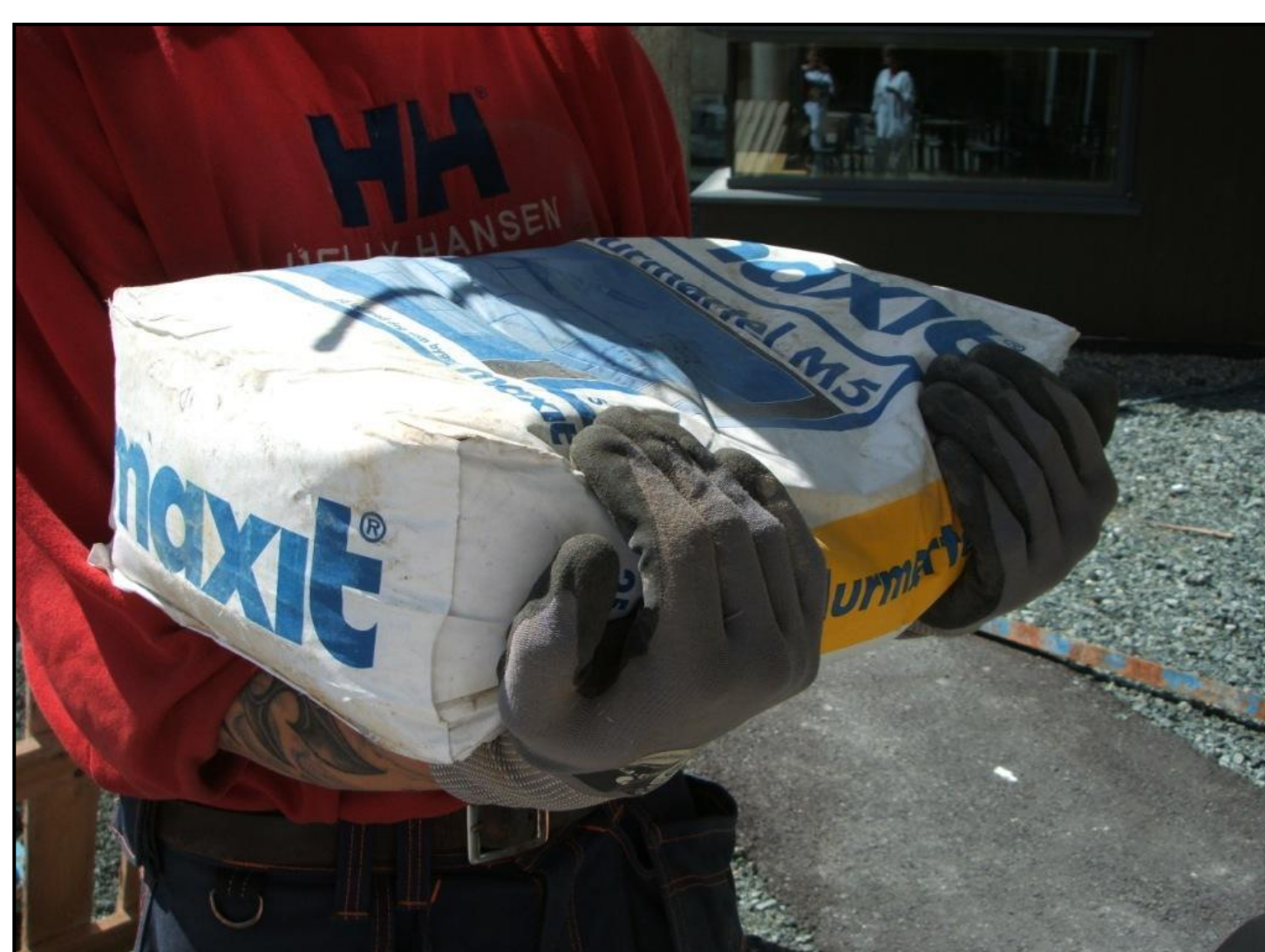

Using the palms of your hands and carrying items with both hands distributes the load across multiple joints.

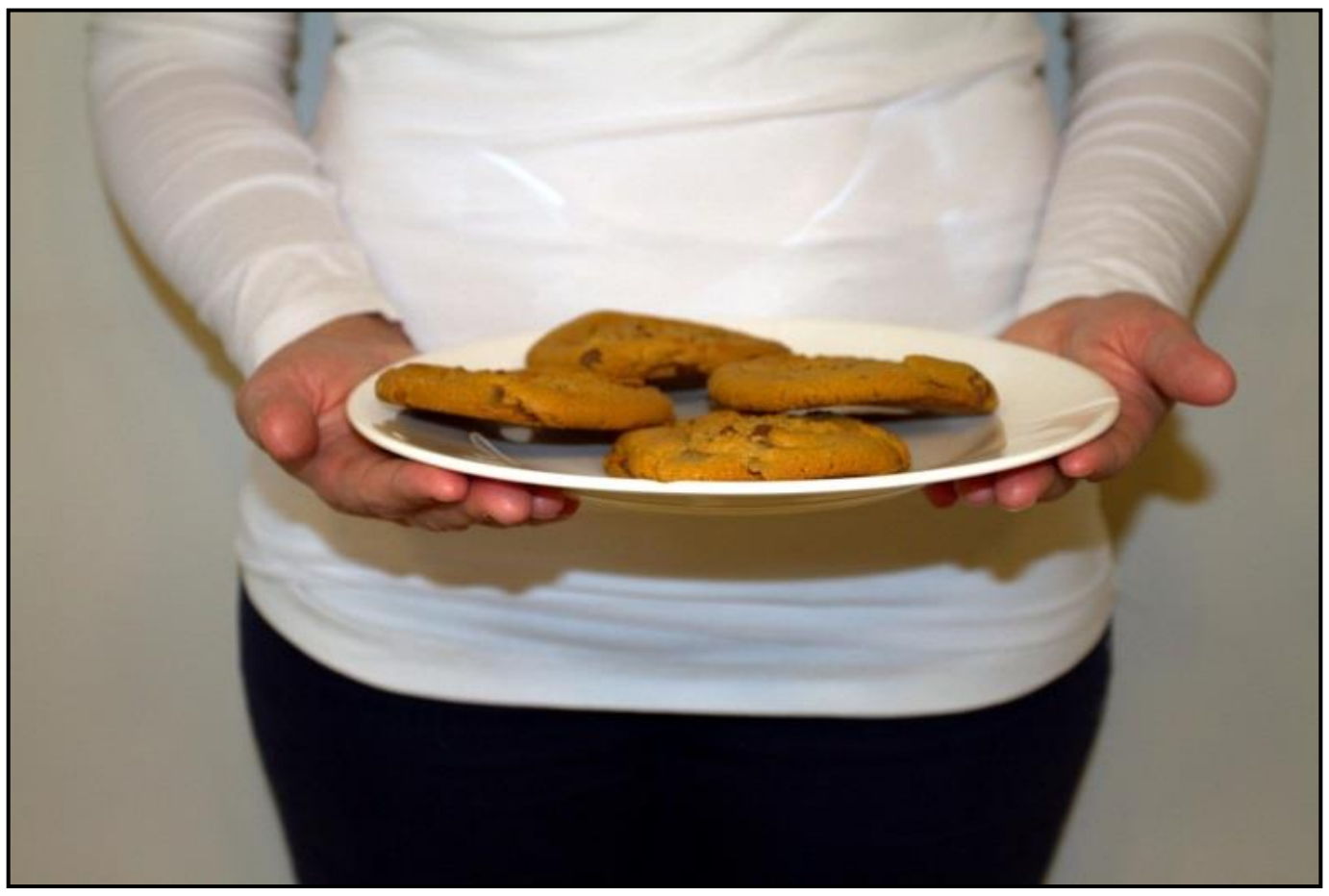

When carrying items with the wrists in neutral position, the joints are stable and hand strength can be used effeicciently.

Shift the load from small to larger joints. These are sorrounded by stronger muscles and can tolerate more stress than the small finger joints.

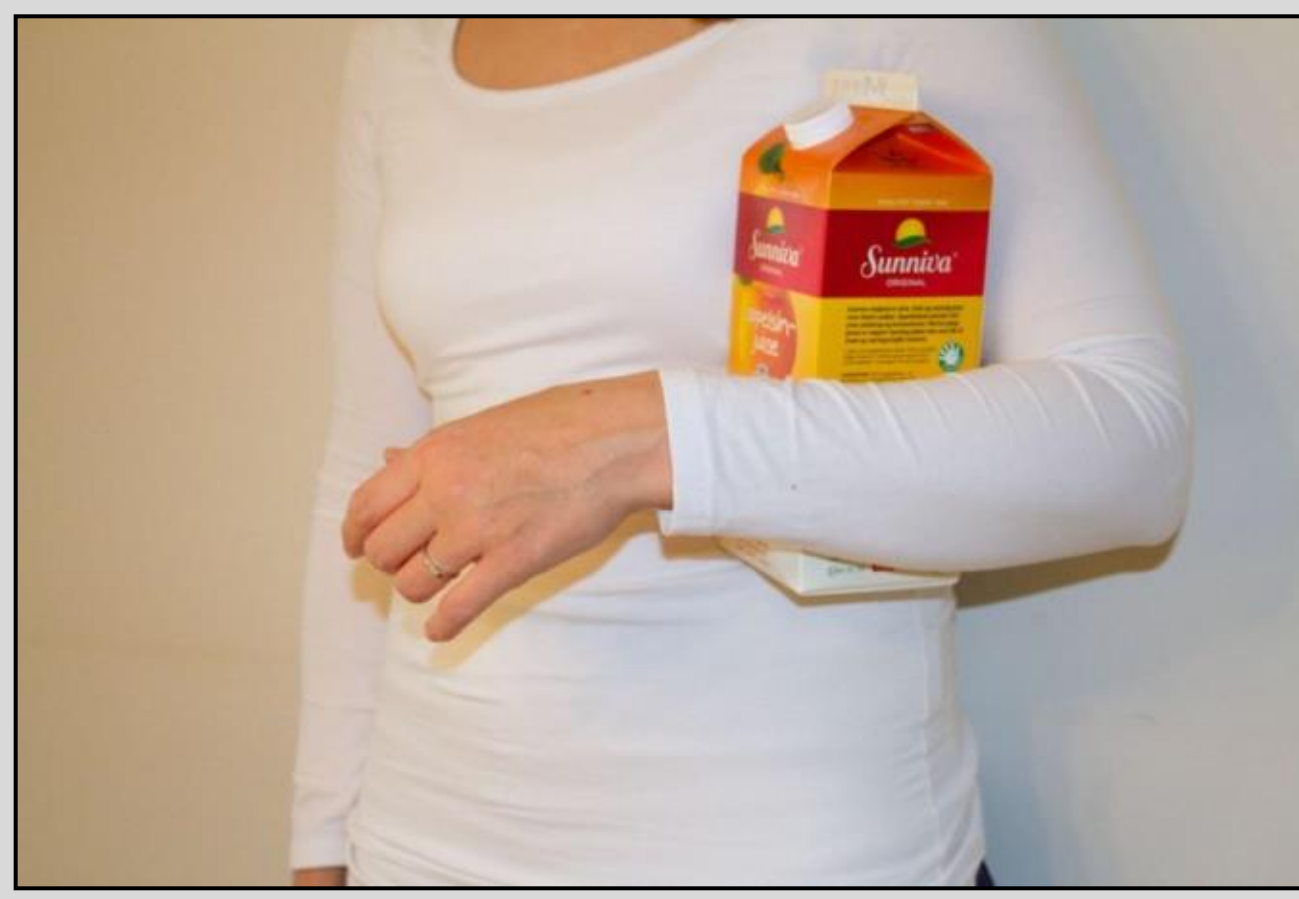

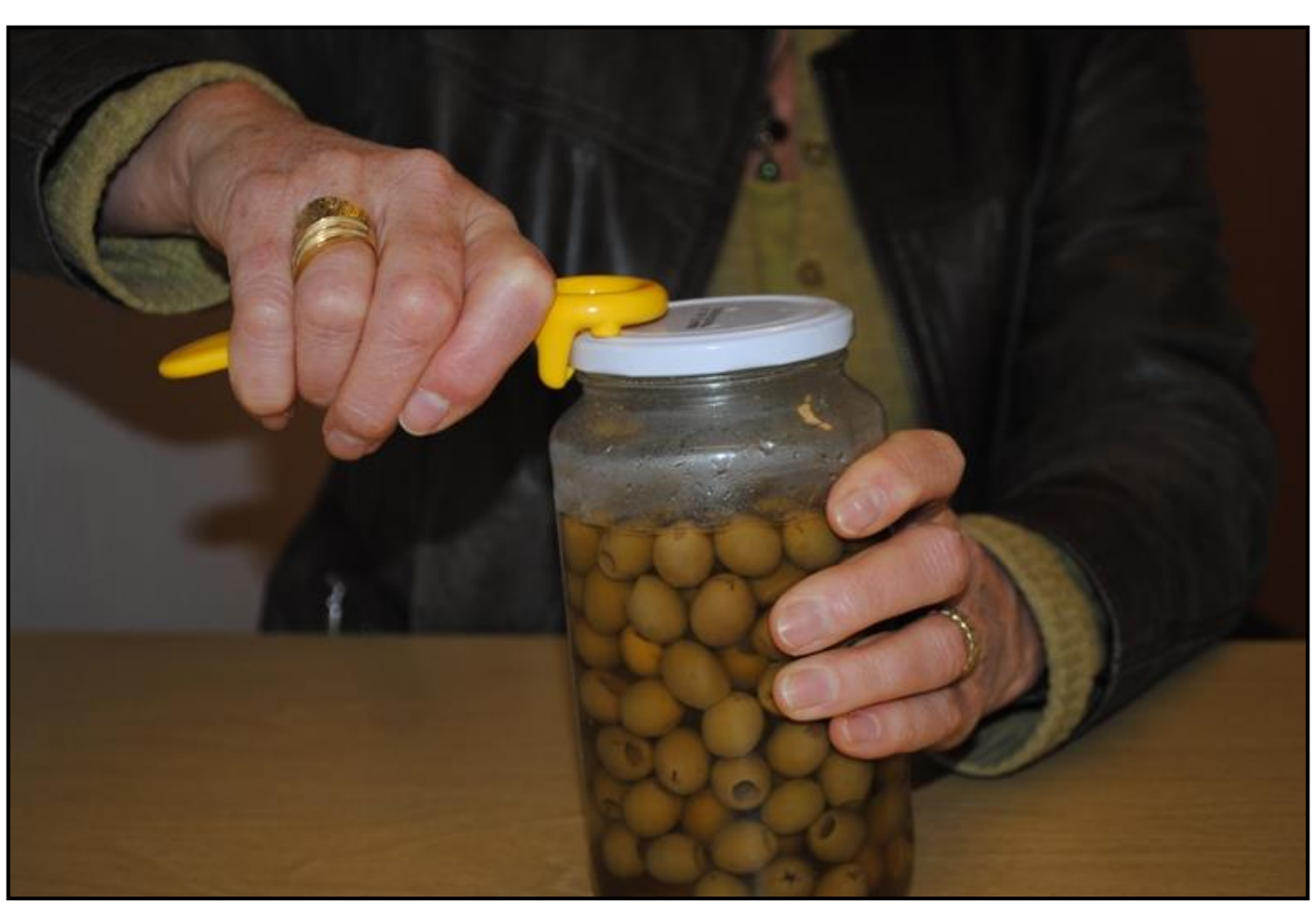

Use equipment that makes tasks easier, such as lightweight frying pans and practical assistive devices.

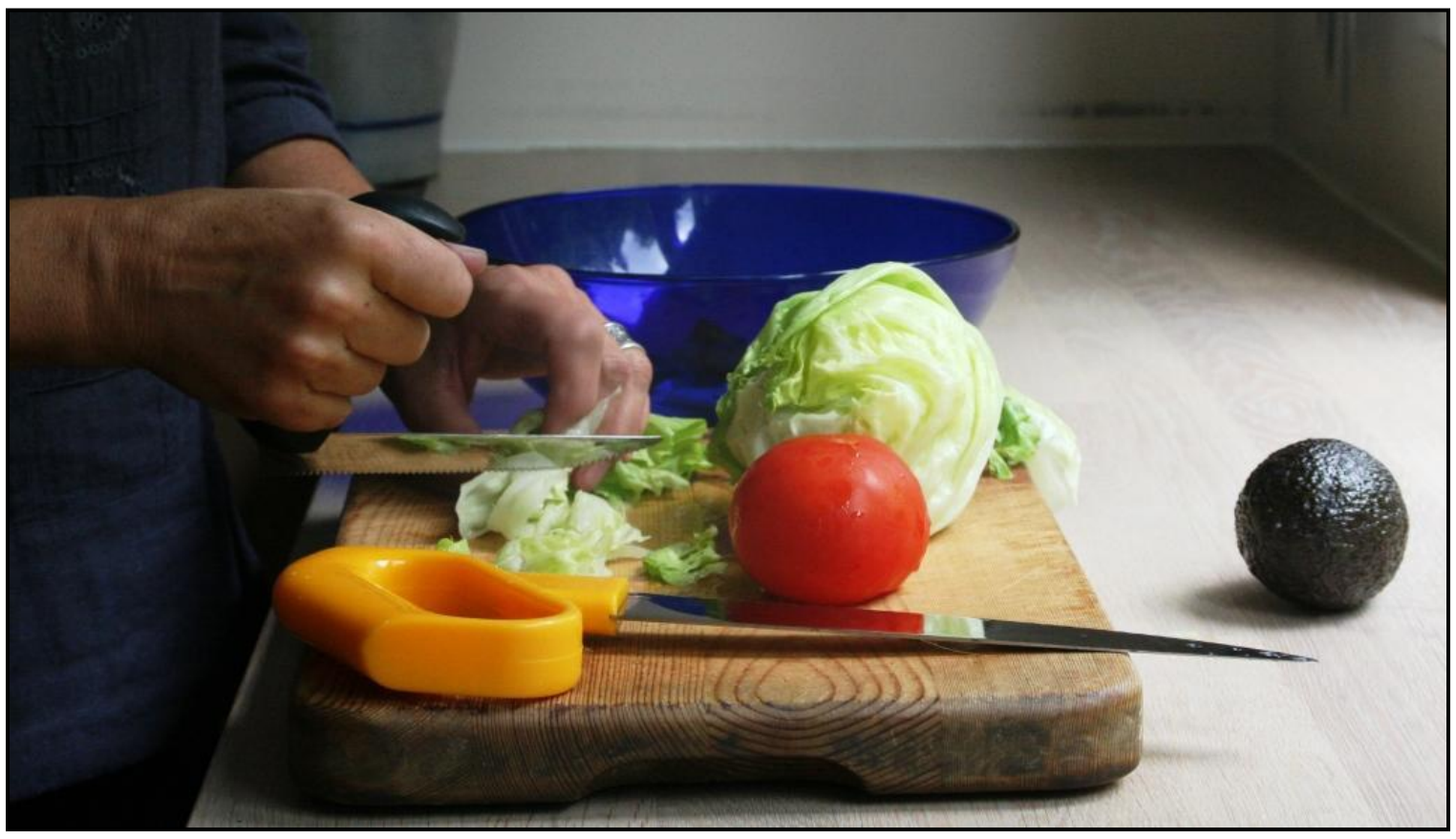

Utensils with thick, angled handles provide a more secure grip and help reduce strain and pain.

Using rubber gloves and an opener for screw top bottles improves grip.

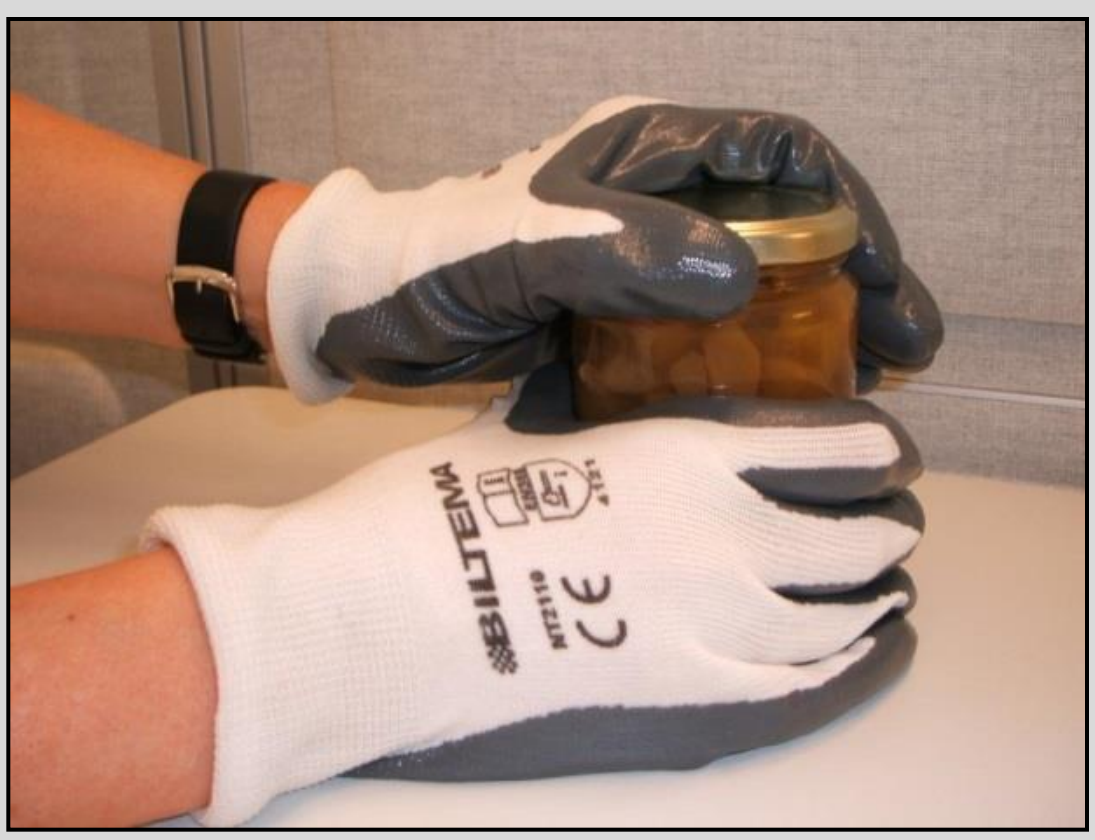

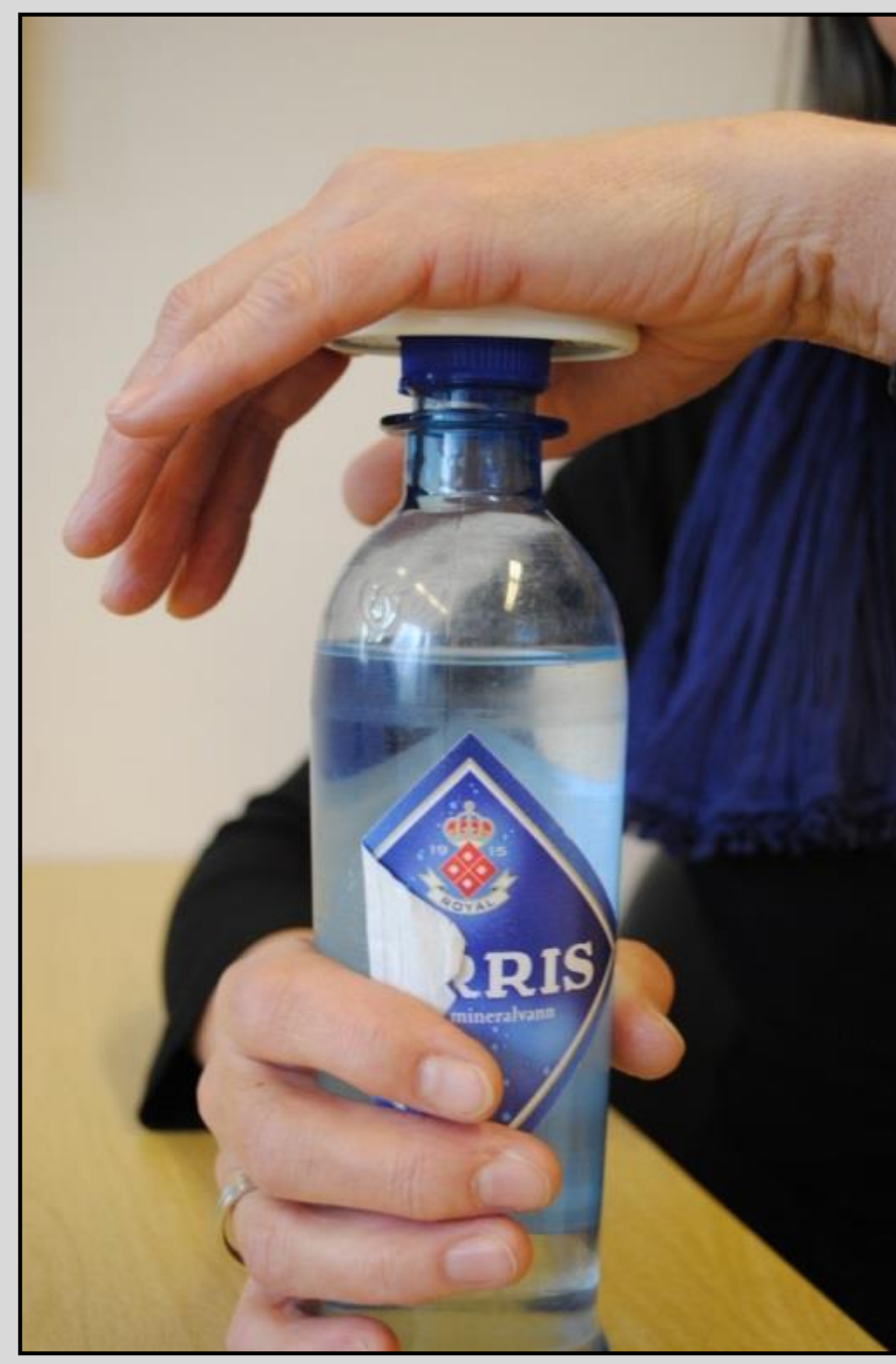

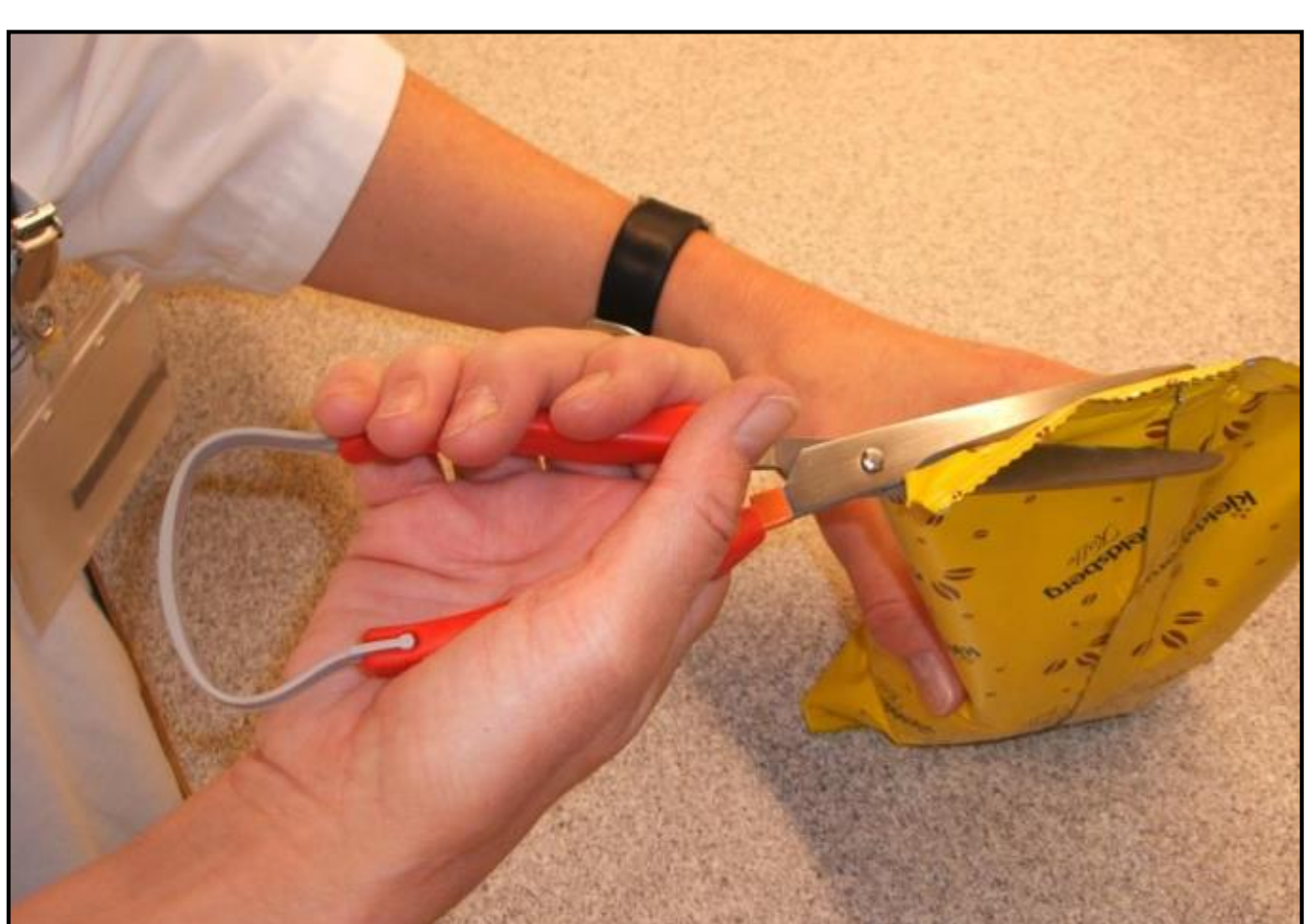

Scissors with spring-loaded handles distribute the load across multiple joints.

ASSISTIVE DEVICES

Assistive devices that makes activity performance easier can be purchased in shops or at the internet. Below we have listed some of these:

- Etac: www.etac.no
- Adl produkcts: www.adl.no
- Ronda: www.ronda.no
- A simpler life: www. enklereliv.no
- Clas Ohlson
- Jernia
- Ikea
- Kitchenware stores
- Pharmacies

.

WHY USE ORTHOSES

If you have osteoarthritis in the thumb base joint, the use of support splints can stabilize the joint so that deformities are prevented and pain relief and improved function are achieved. Research shows that using a rigid support splint at night over time reduces pain. Furthermore, using a smaller support splint during the day can make it easier to perform strenuous activities when you have painful or unstable joints

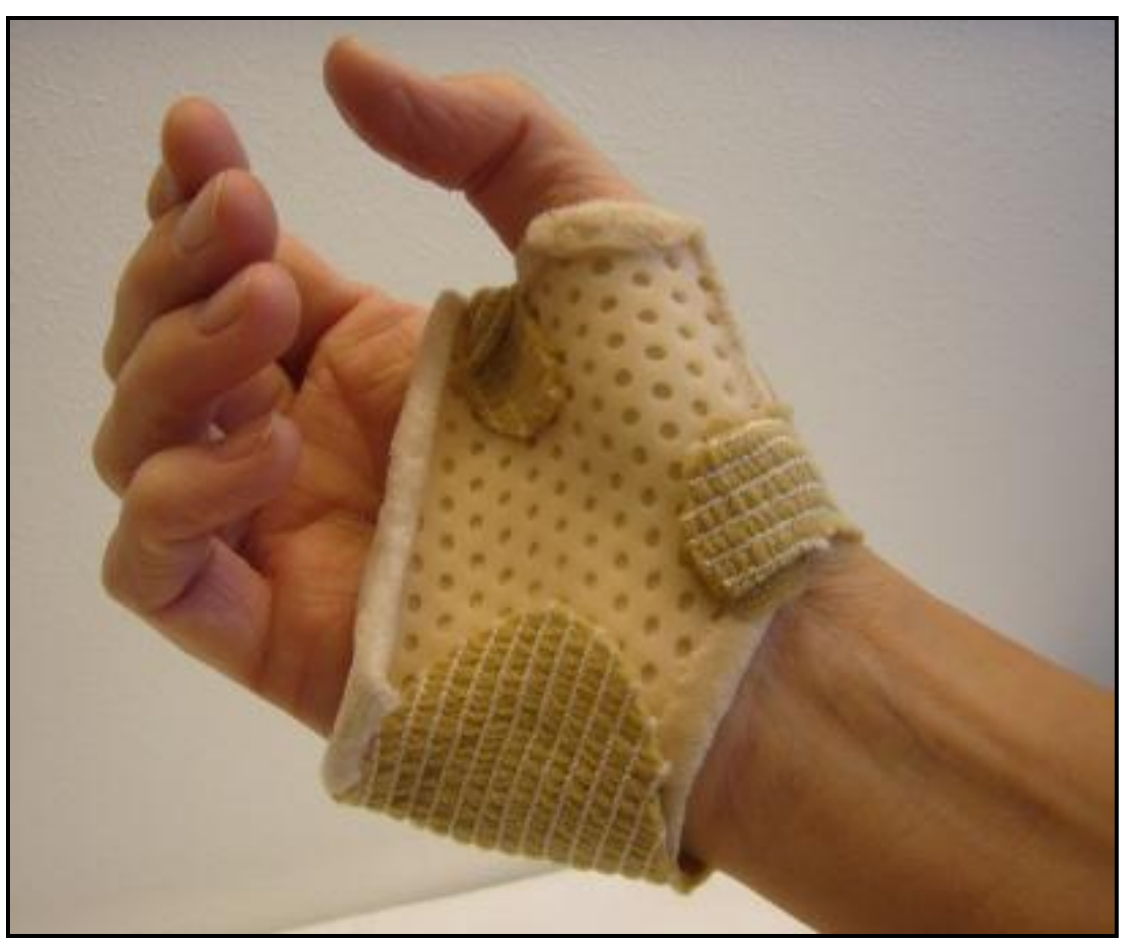

The best type of day orthosis varies from person to person.

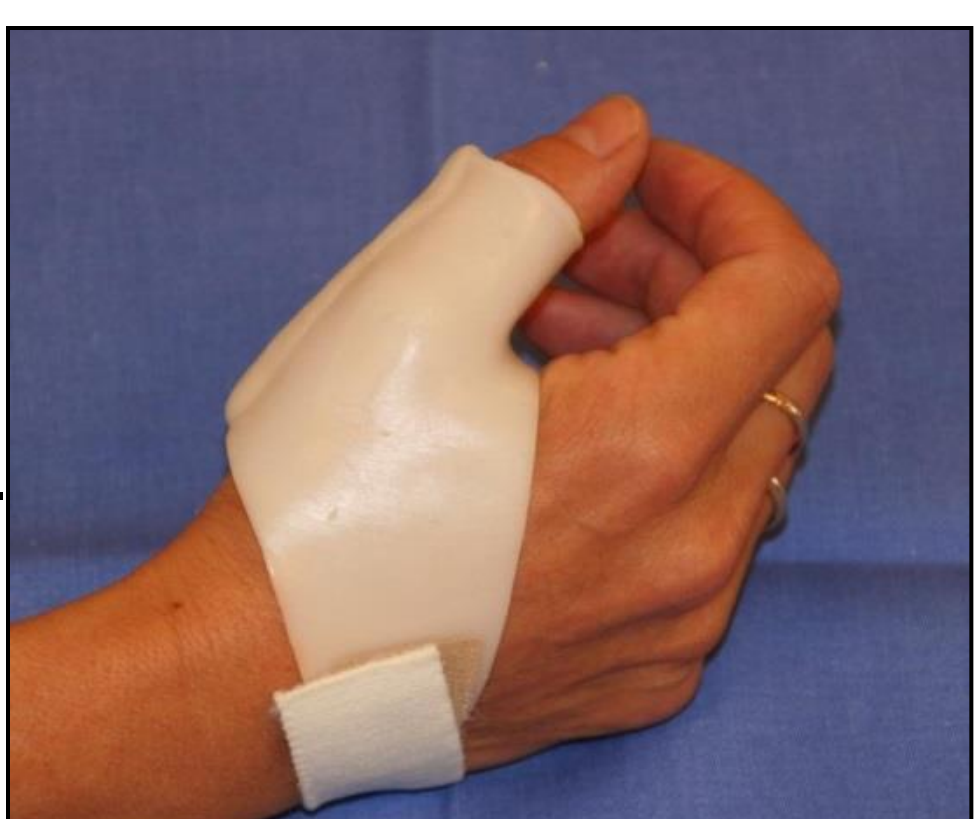

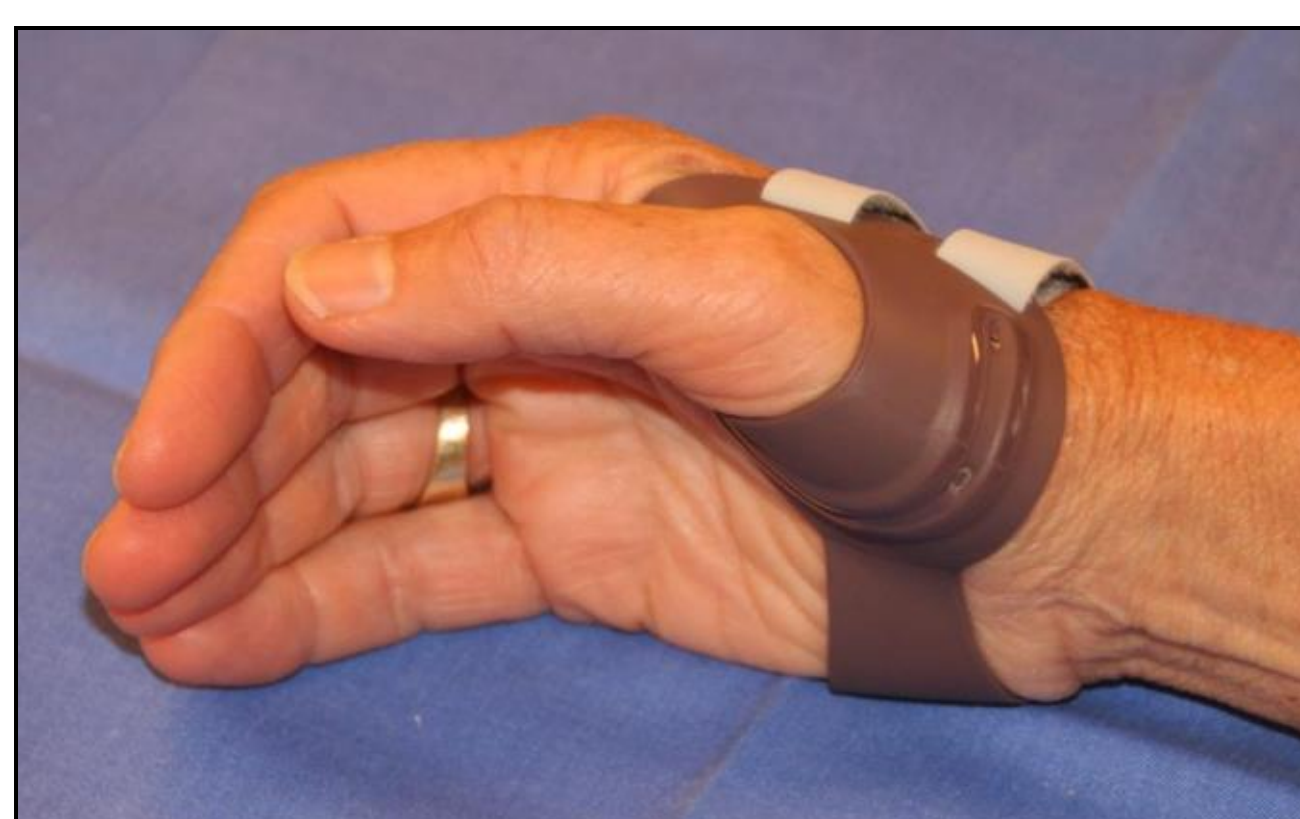

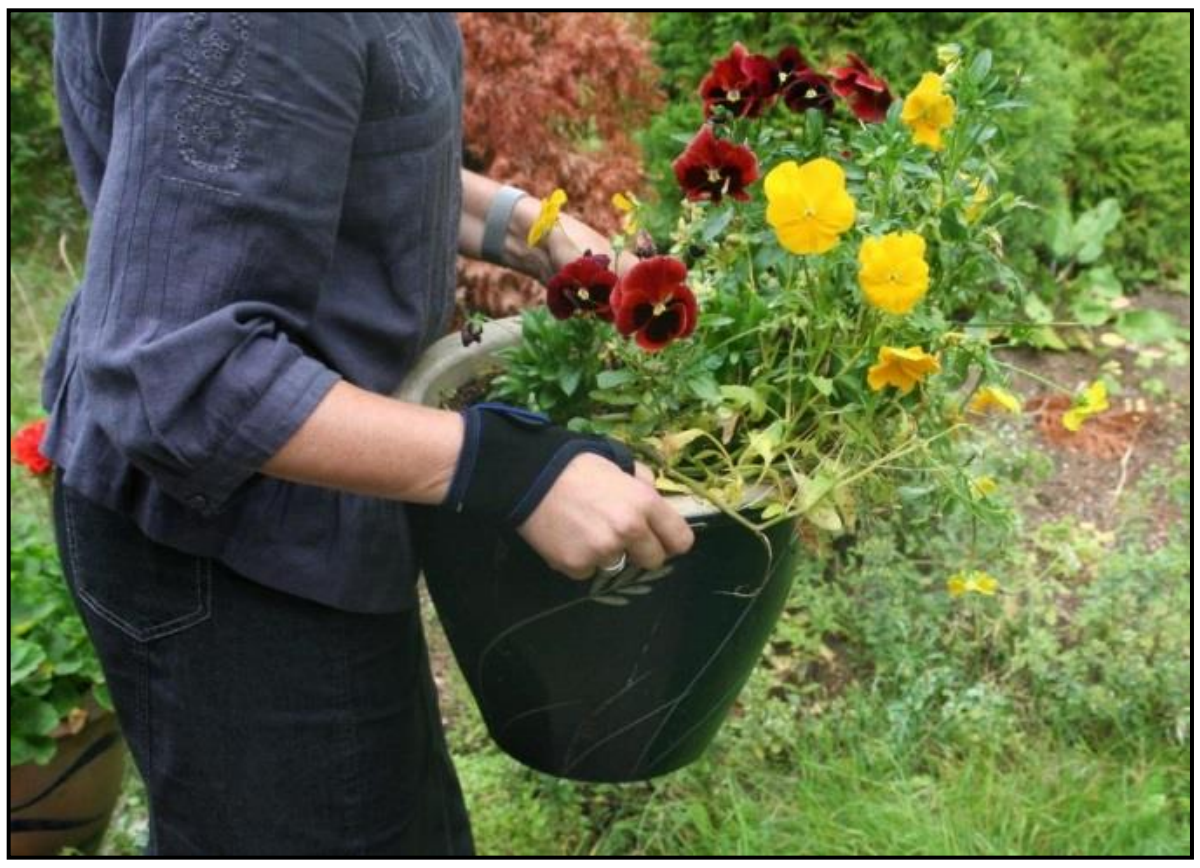

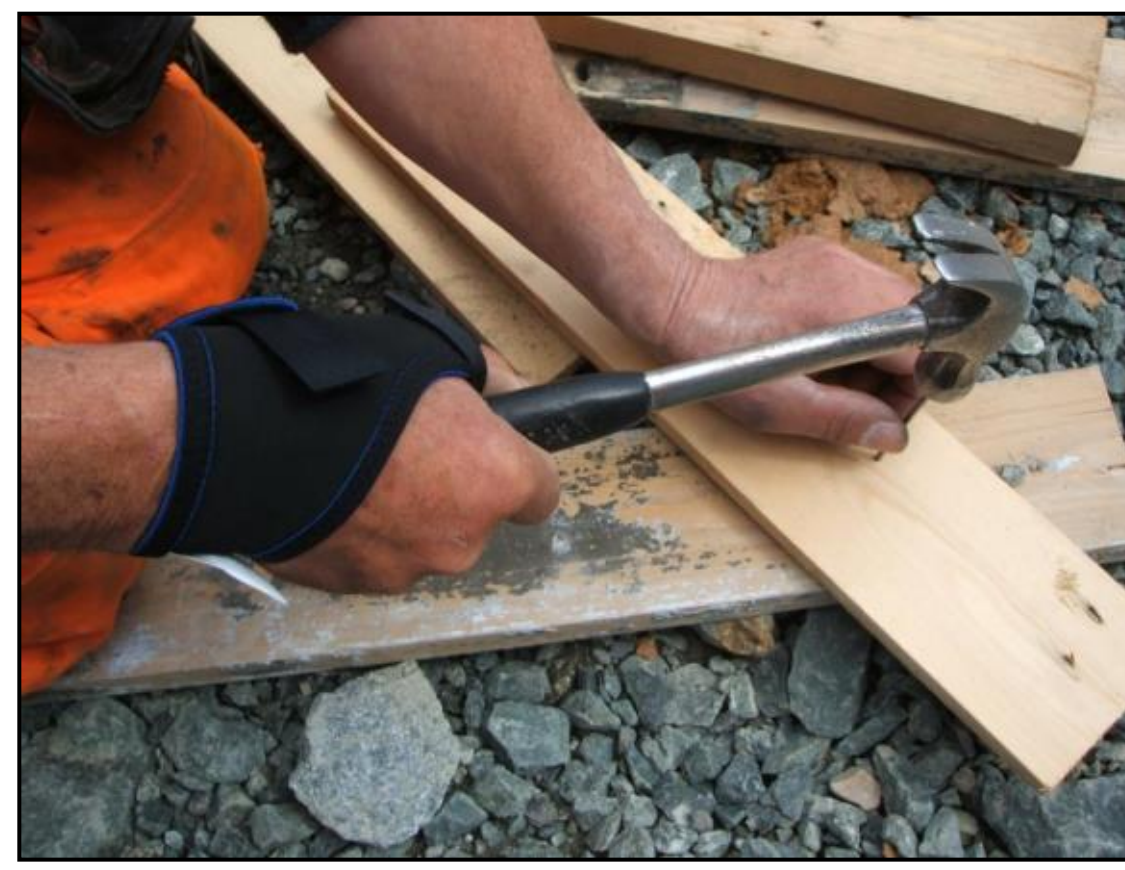

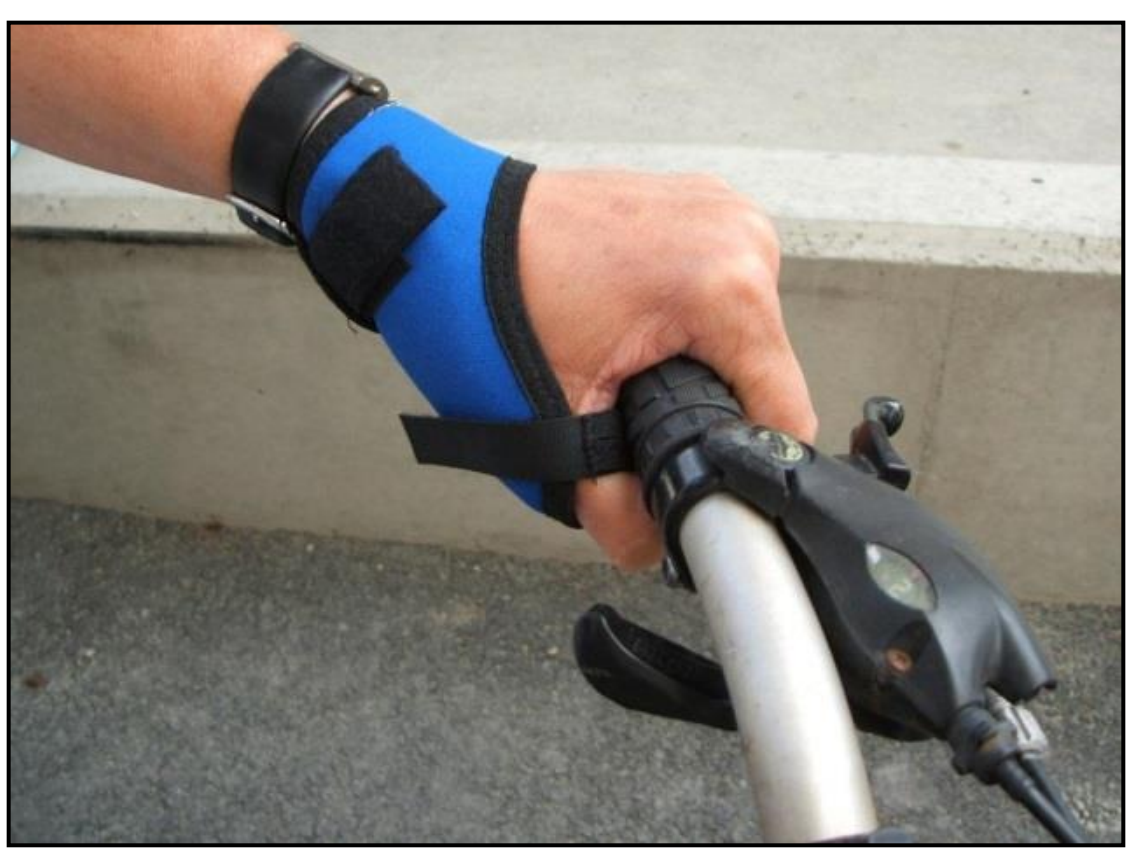

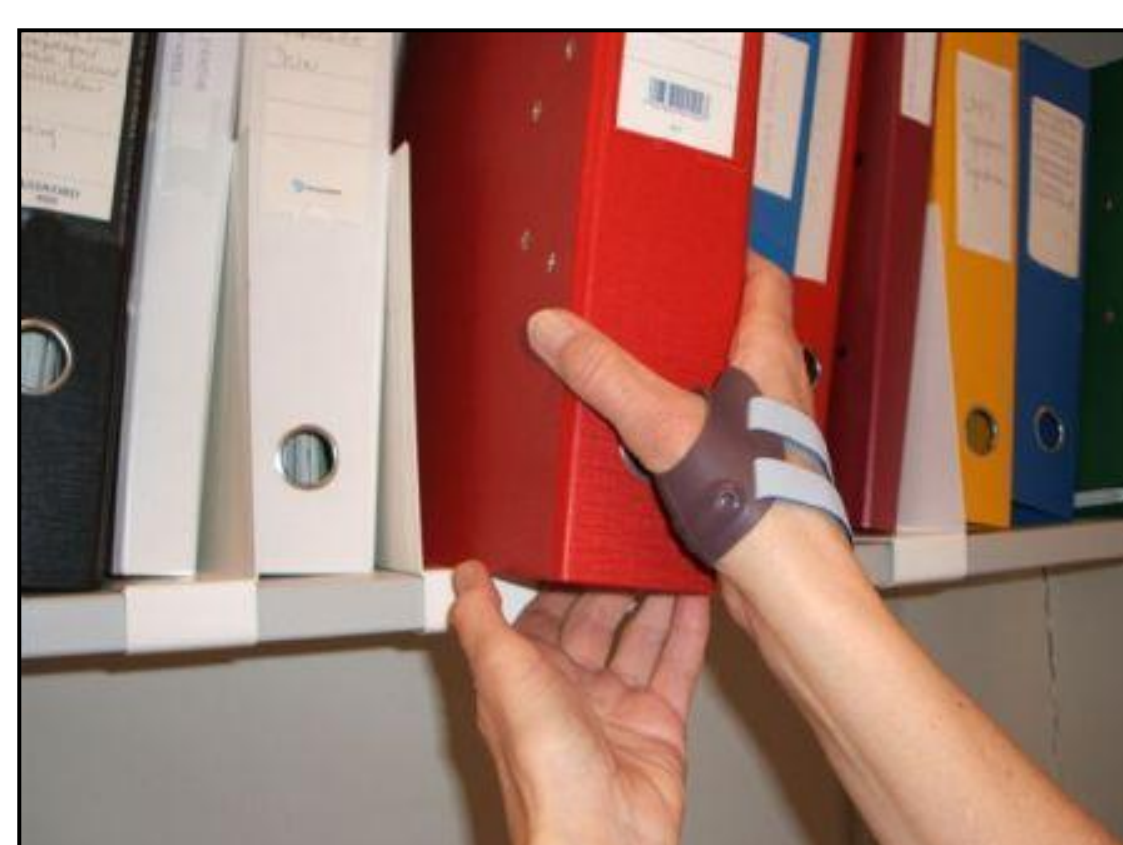

HAND EXERCISES

Osteoarthritis cannot currently be cured. The aim of treatment is to achieve pain relief, improve function, and slow the progression of the disease in the joints. Movement and use of the body improve the nourishment of the cartilage and strengthen the joints. Research shows that exercise to maintain mobility and strengthen the muscles can reduce pain and improve physical function in the long term.

The exercise program

The exercises can be done anytime and anywhere, but you should concentrate on the task and perform them correctly. Many people find it easier to complete the exercises at a fixed time, and preferably also in a fixed place. Sit at a table or use the armrests of a chair so that the forearms are well supported on the surface. Make sure to lower and relax the shoulders before you start and breathe well throughout.

Before starting the exercises, you can warm up by applying moisturizing cream to the hands and massaging it well in. Most exercises are performed five times during the first two weeks. After that, you can increase to ten repetitions and continue at that level. We recommend exercising 2–3 times per week.

It is not uncommon to feel some aching afterwards, especially during the first sessions. However, this will subside as the joints and muscles become more flexible and stronger. If the pain persists, the training should be adjusted for a period and then gradually increased again once the pain has settled.

On the following pages, you will find an exercise program adapted for hand osteoarthritis. A video of the exercises is also available on www.youtube.com (search for “håndartrose” and “håndtrening”).

Hand exercises for joint mobility

1. Thumb mobility: Keep the thumb IP and MCP joints slightly flexed throughout. First, open the hand as if grabbing a bottle. Bring the index fingertip to the thumb tip, keeping the MCP, PIP and DIP joints flexed. Open the hand again ("grab the bottle"). Repeat with the 3rd, 4th and 5th fingers. Start with five repetitions and increase to ten over the next two weeks.

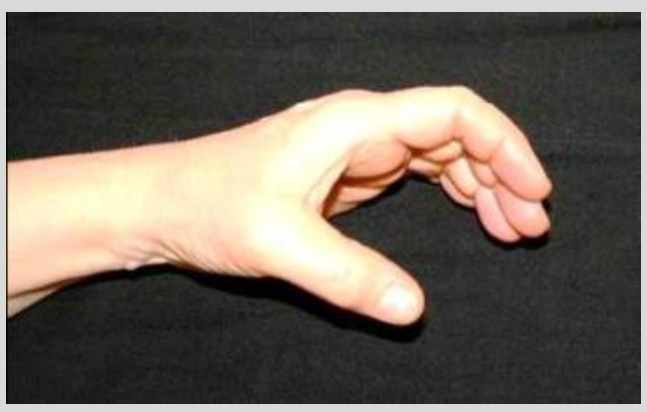

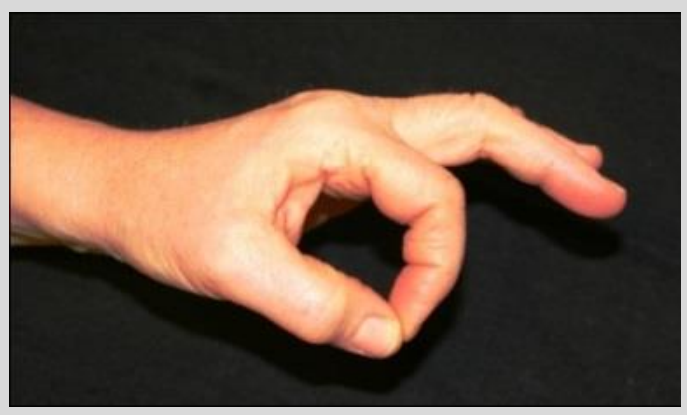

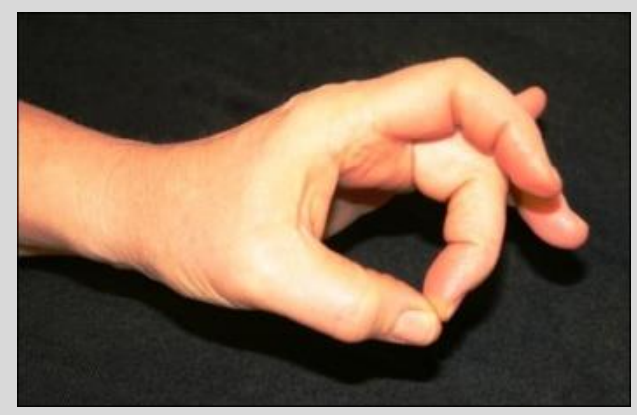

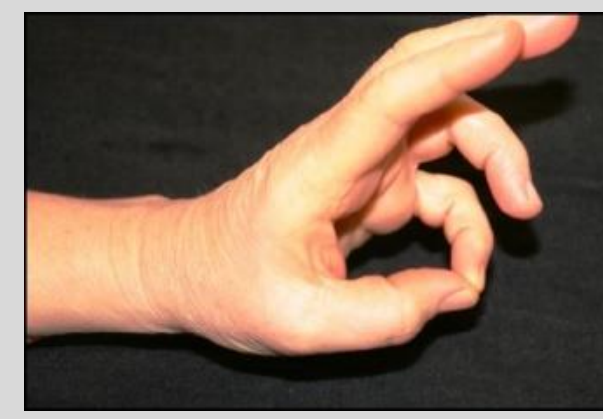

2. Finger mobility: First, flex the 2nd to 5th DIPs and PIPs only (keep the MCPs extended). Then flex the MCPs. Hold for 5 seconds. Reverse: extend the MCPs only, then the PIPs and DIPs. Start with five repetitions and increase to ten over the next two weeks.

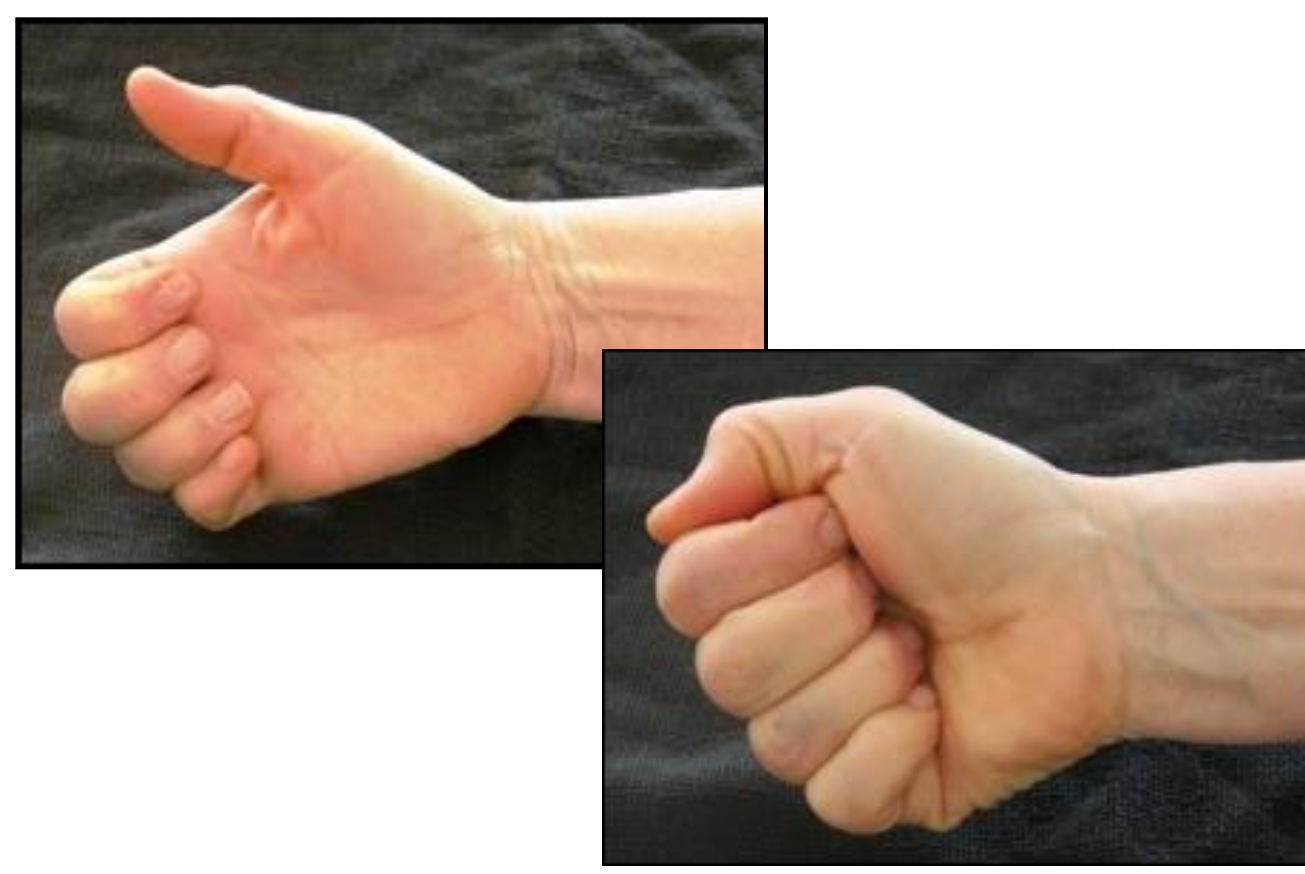

Hand exercises for stability and strength

3. Thumb stabilizer: (do not do this exercise if you have strong pain in your thumb base joint). Rest your hand rest on a surface. Place a rubber band in figure eights around your thumb just below the outer joint and around the fingers as shown in the picture (between the PIP and MCP-joints). Keep the MCP and IP joints of the thumb flexed while abducting/extending the thumb. The abducted position is maintained for 5 seconds. Start with five repetitions and increase to ten. As you are getting stronger, you may also change to a thicker rubber band, or you may use two or more thinner bands together.

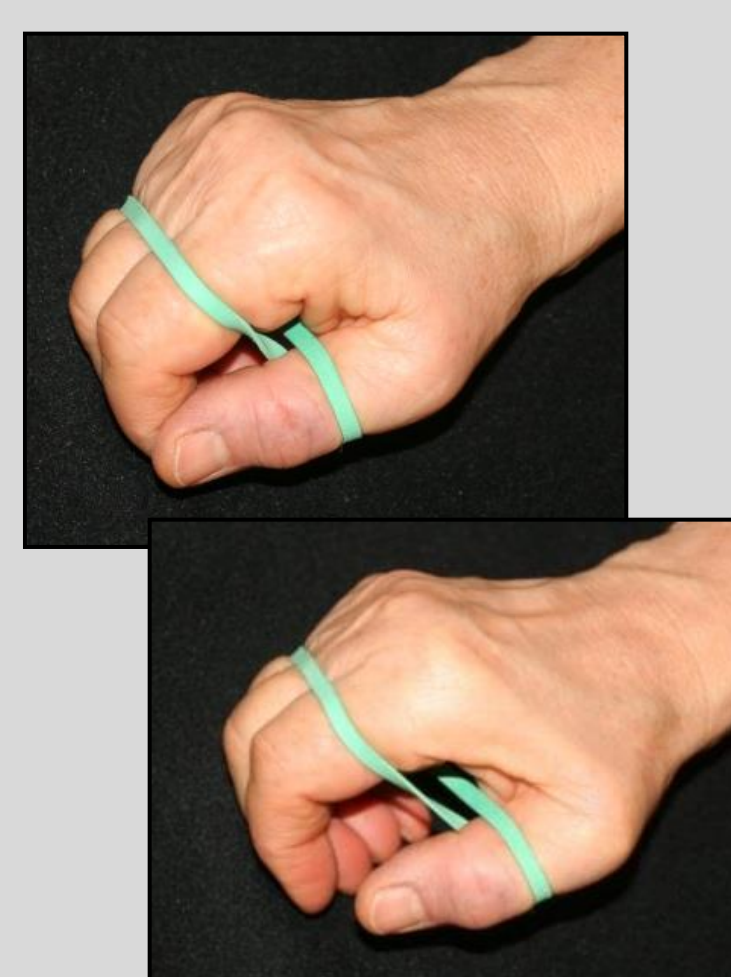

4. Grip strength: Squeeze around a pipe insulation tube or a ball with maximum force (as hard as you can) and release. Start with ten repetitions and reduce to five.

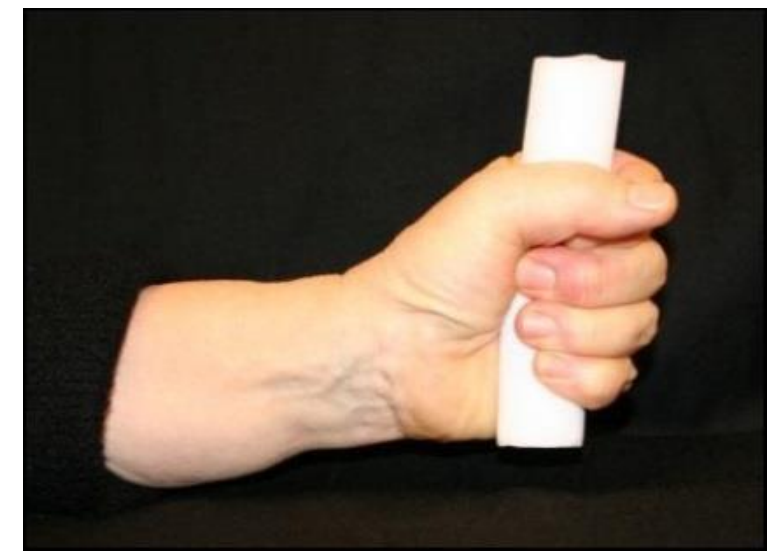

5. Wrist stability: Hold an exercise band between your hands and pull it apart. The wrists should be kept still in a neutral position while performing the exercise (as shown in the picture). Pull apart – hold for 5 to 10 seconds, then release. Start with 5 repetitions and gradually increase to 10. **Note:** Keep the shoulders lowered during the exercise.

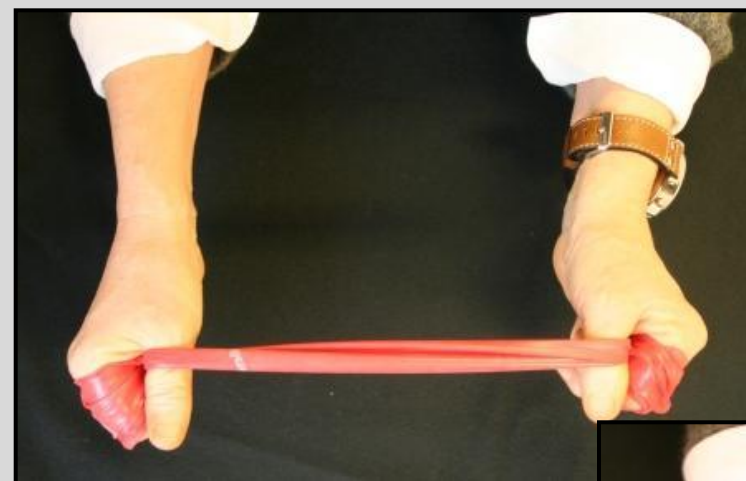

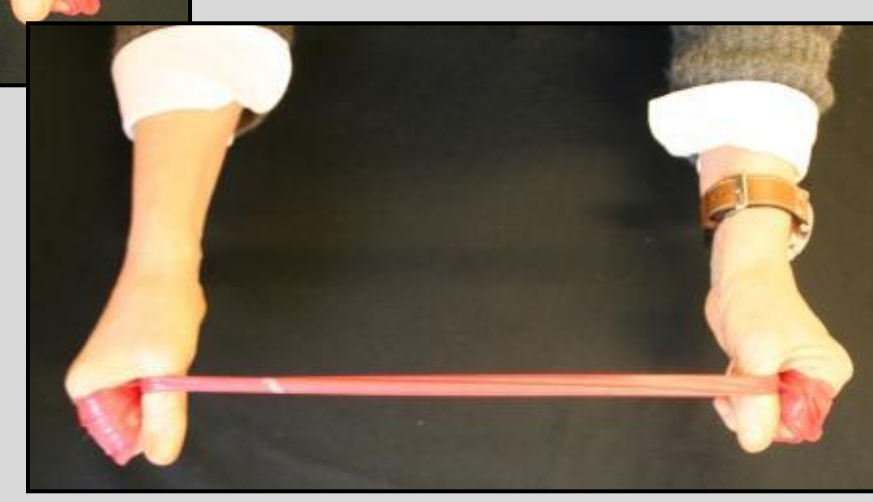

Stretching

6. Thumb stretching: Spread all the fingers, including the thumb, and hold for 30 seconds. Then move the fingers together again. Repeat three times.

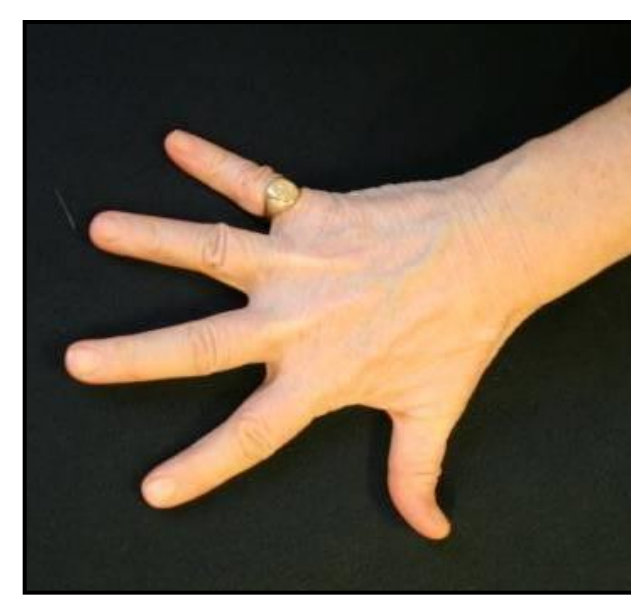

7. Finger stretching: Put your hand on the table with the palm facing down. Put your other hand on top and press the PIP and DIP-joints down against the table. Hold for 30 seconds. Repeat three times.

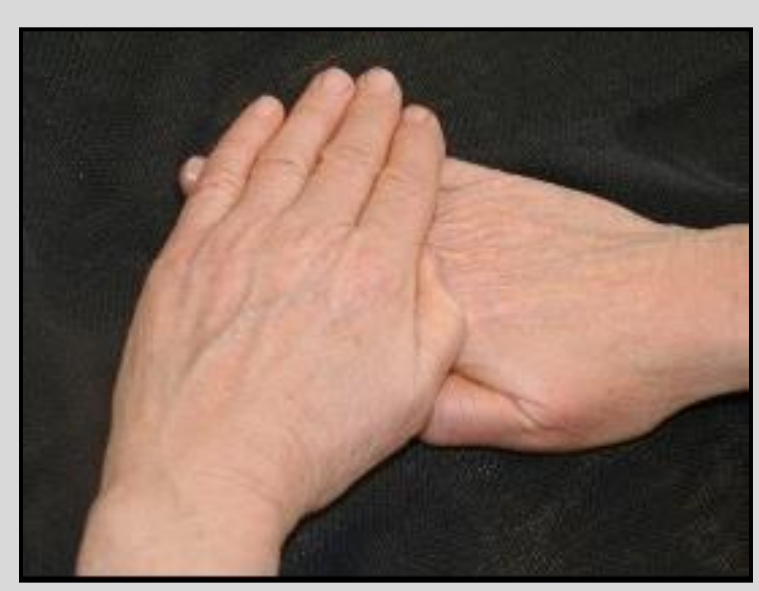

Alternative finger stretching: If direct pressure against the joints are painful, put one finger at each side of the PIP and DIP joint and push down against the table. Repeat for each finger.

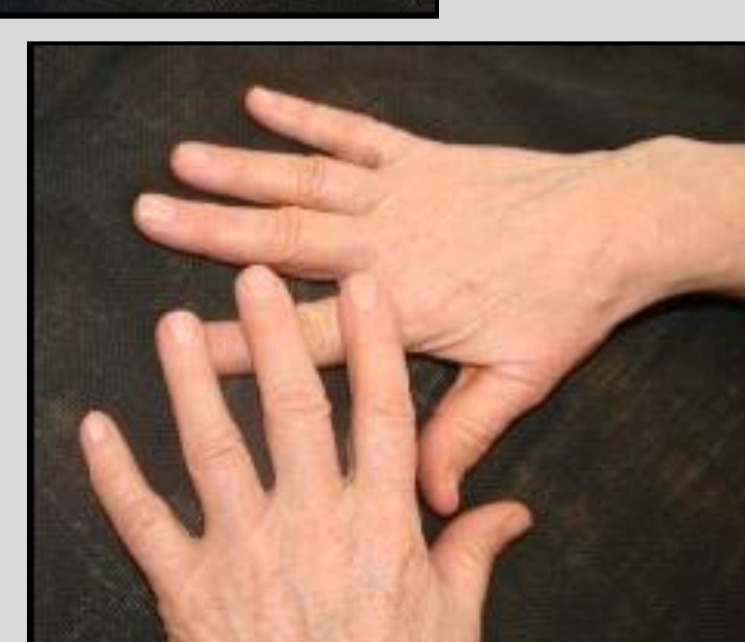

Supplement: Supplementary file 1 [file mmc1.pdf]
